# The Effectiveness of Health-Oriented Interventions and Health Promotion for Unemployed People—A Meta-Analysis

**DOI:** 10.3390/ijerph20116028

**Published:** 2023-06-01

**Authors:** Karsten Ingmar Paul, Alfons Hollederer

**Affiliations:** 1 School of Business, Economics, and Society, Friedrich-Alexander-Universität Erlangen-Nürnberg, 90403 Nürnberg, Germany; 2 Department of Social Work and Social Welfare, The Faculty of Human Sciences, University of Kassel, 34127 Kassel, Germany

**Keywords:** unemployment, health, mental health, health promotion, exercise, cognitive behavioral therapy, job search, meta-analysis

## Abstract

*Background:* Unemployment is known to have negative effects on mental and physical health. Yet, the effectiveness of interventions aimed at improving the health of unemployed people is unclear. *Methods:* We conducted a random-effects meta-analysis of extant intervention studies with at least two measurement points and a control group. A literature search in PubMed, Scopus, and PsycINFO in December 2021 identified 34 eligible primary studies with 36 independent samples. *Results:* For mental health, the average meta-analytic effect sizes for the comparison of the intervention group and the control group were significant and of small size after the intervention, *d* = 0.22; 95% CI [0.08, 0.36], as well as at follow-up, *d* = 0.11; 95% CI [0.07, 0.16]. Effects on self-assessed physical health status were small and marginally significant (*p* = 0.10) after the intervention: *d* = 0.09; 95% CI [−0.02, 0.20], and insignificant at follow-up. However, when job search training was not part of the intervention program (i.e., all available resources were used solely for health promotion), the average effect size for physical health was significant after the intervention, *d* = 0.17; 95% CI [0.07, 0.27]. Furthermore, the effects of physical activity promotion were significant and of small-to-medium size after the intervention, leading to increased levels of activity, *d* = 0.30; 95% CI [0.13, 0.47]. *Conclusions:* Population-based health promotion programs are recommended because even measures with small effect sizes can actually improve the health of a large group of unemployed people.

## 1. Introduction

The pandemic caused by the SARS-CoV-2 virus resulted not only in a direct threat to public health due to illness but also led to disturbances in the labor market. Specifically, there was a strong initial rise in unemployment rates during the early phases of the pandemic in several OECD countries, with youths, people with low education, and people in low-paying occupations being particularly severely hit. In later stages, this was followed by a rise in the number of people who were unemployed for more than six months and may end up in long-term unemployment [1,2].

There is ample empirical evidence that unemployment has negative effects on the health of the individuals experiencing it. Meta-analyses demonstrated a negative impact of unemployment on mental health [3,4,5], with several different aspects of mental health being affected. For example, unemployed people report more depression symptoms, more symptoms of anxiety, reduced life satisfaction, and impaired self-esteem compared to employed individuals [5]. In line with this, unemployment also increases the risk of suicide, even when preexisting mental health problems are controlled [4]. The self-reported general health of unemployed people is also worse than that of employed people [6,7,8]. Finally, the prevalence of various specific diseases such as bronchial asthma, diabetes, and some forms of cancer and cardiovascular diseases appears to be elevated among unemployed people compared to employed people [9]. As a result of these multiple differences in health, the mortality rate of the unemployed is 63% higher than that of the employed [10]. Yet, despite the obvious importance of mental and physical health for unemployed people, there are relatively few intervention studies that explicitly aim to improve the health of unemployed people. 

The aim of the present investigation was to identify existing intervention studies that tried to improve the health of unemployed people and to integrate these studies meta-analytically. This first thorough meta-analysis on this topic aims at answering several important questions, for example: (1) Are health-related interventions for unemployed people generally effective? (2) How strong is the average effect size of such interventions? (3) Which types of interventions are more effective than others? (4) Are the interventions more effective for certain subgroups of participants (e.g., long-term unemployed people)? Furthermore, the validity of the findings will be checked through an extensive sensitivity analysis. 

### 1.1. Approaches to Improve the Health of Unemployed People

The existing interventions aimed at improving unemployed people’s health are diverse in content and form. Many are focused on mental health, using psychological methods such as cognitive-behavioral training [11]. Others incorporate interdisciplinary case-management approaches [12], skills training [13], or counseling for physical exercise [14]. They include elaborate and highly structured programs using several different established techniques simultaneously [15] as well as interventions relying on a single specific method, such as progressive muscle relaxation [16]. Their duration ranges from a single one-hour session [17] to programs lasting up to two years [18]. They target young unemployed people [19] as well as people in the late stages of their working lives [12], and short-term [15] as well as long-term unemployed people [20]. 

Yet, despite this diversity, the large majority of existing health-oriented interventions for unemployed people are based on well-established theoretical approaches, such as cognitive behavioral therapy [21], motivational interviewing [22], action theory [23,24], or social-cognitive theory [25]. 

There are three earlier systematic reviews on the topic of health-related interventions for unemployed people [26,27,28]. These reviews provided some very interesting insights but, being qualitative syntheses, did not conduct a quantitative integration of the research field’s main findings. 

Since the methods applied in health-oriented interventions for unemployed people have successfully been used in other health-related contexts, we expect the existing interventions for unemployed people to be effective, particularly for mental health symptoms but also for physical health and health behavior.

**H1:**  *Health related interventions for unemployed people have a positive impact on (a) mental health, (b) physical health, (c) health behavior.*

### 1.2. Factors Moderating the Effectiveness of Health-Related Interventions for Unemployed People 

Up to now, it is not clear whether certain methods used to improve the health of unemployed people are more successful than others, whether specific context variables (e.g., the amount of training received by each participant or the duration of the training program) positively or negatively influence the effectiveness of interventions, or which subgroups of unemployed people profit most from participation in an intervention. Knowledge about such moderators would be very valuable, however, for decisions on how exactly the limited resources that are typically available for health-related interventions for this group of people should be used. 

#### 1.2.1. Characteristics of the Intervention as Moderators of Intervention Success

##### Type of Intervention

Several different types of interventions have been developed or adapted for the field of unemployment research. At least some of them have a high likelihood of success in our eyes. 

*Cognitive methods*

Cognitive-behavioral methods aim to improve health by changing maladaptive cognitions and behaviors [29]. Thus, mental health is improved by changing how people think about themselves and their lives. These methods are among the most effective in psychotherapy [30]. They have also been successfully implemented to change health behavior [31].

**H2:**  *Interventions including cognitive-behavioral methods will be more effective in improving unemployed people’s health than interventions not including such methods.*

*Non-cognitive stress-management techniques*

Some studies have employed stress-management methods that cannot be categorized as typical cognitive-behavioral, for example, stress inoculation [15,32], assertiveness training [33], or positive psychology interventions [34]. Since these interventions have been successfully used in other contexts, we expect this relatively heterogenous category of interventions to also be successful with unemployed people.

**H3:**  *Interventions including non-cognitive stress management methods will be more effective in improving unemployed people’s health than interventions not including such methods.*

*Relaxation techniques*

Relaxation therapy comprises a range of different techniques, such as progressive muscular relaxation, autogenic training, mindfulness-based therapy, and meditation [35]. They all share the assumption that relaxation can be used to treat different forms of distress and other ailments. Endorsing this assumption, relaxation has been shown to be effective not only in the treatment of anxiety [36] but also for other psychological disorders such as depression [37,38].

**H4:**  *Interventions including relaxation techniques will be more effective in improving unemployed people’s health than interventions not including relaxation techniques.*

*Strengthening of social support*

In some interventions for unemployed people, an increase in the amount of social contact and an improvement in the social support that is available for the participants are used as means to strengthen their health. Social support has repeatedly been shown to be generally beneficial for different aspects of health [39]. Furthermore, social support buffers the negative effects of stressors [40]. Since being unemployed is a very stressful life situation, individuals in this situation are likely to profit from the buffering effect of social support [41].

**H5:**  *Interventions including methods to strengthen the social support that is available to participants will be more effective in improving unemployed people’s health than interventions not including such methods.*

*Job search training*

Several intervention studies have aimed at simultaneously increasing their unemployed participants’ mental health as well as improving their job search skills. Insofar as such a training leads to a more hopeful and optimistic outlook regarding one’s chances of finding a new job, it might alleviate distress. In line with this reasoning, reemployment expectations have been shown to be positively correlated with unemployed people’s mental health [3]. However, strong job-search efforts and a strong work centrality—two variables that might also receive a boost during job-search training—are negatively associated with mental health among the unemployed [3,5]. We therefore abstain from proposing a specific hypothesis and instead formulate a research question. 

Research question 1: Does the inclusion of a form of job search training in an intervention for unemployed people influence their mental or physical health?

*Physical exercise*

A minority of health-related interventions for unemployed people have also included sessions of physical exercise in their programs. Since unemployed people show a tendency towards unhealthy lifestyles, including high smoking rates and sedentary behavior [42,43], the direct inclusion of exercise might be a helpful amendment to programs targeting unemployed individuals’ health. Generally, physical activity has positive effects on different aspects of physical health [44], as well as mental health [45,46].

**H6:**  *Interventions including physical activity will be more effective in improving unemployed people’s health than interventions not including physical activity.*

*Health-related counselling*

A minority of studies have included counseling concerning physical health in their intervention program, which might, for example, consist of a questionnaire-based diagnosis of the participant’s level of health behavior, followed by an individualized counseling session in which goals for improving the participant’s health behavior are developed and possible methods to achieve these goals are suggested. Since counseling about health behavior has been shown to be effective for different types of patients and different kinds of health behavior (e.g., [47,48,49]), it will probably also improve the respective behavior among unemployed people. Furthermore, insofar as such an intervention increases participants’ health-related self-efficacy and optimism, it might also improve psychological well-being.

**H7:**  *Interventions including health counseling will be more effective in improving unemployed people’s health than interventions not including health counseling.*

##### Formal Characteristics of the Intervention 

*Number of participants per group*

Health-related interventions for unemployed people are usually provided in group settings, which leads to the question of whether the size of the groups has an influence on the program’s effectiveness. Large groups might offer more kinds of social contact and more opportunities for social support. However, a large number of participants per group could also lead to a dilution of the individualized attention each group participant receives from the trainer, possibly reducing the effectiveness of the intervention. Larger groups might also produce more disruptions and distractions, impeding their effectiveness. In line with this reasoning, research on the influence of class size on the academic achievement of students has demonstrated a negative association, with larger classes leading to worse achievement than smaller classes [50], although the effect might be rather small [51].

**H8:**  *The higher the number of participants per training group the smaller is the effectiveness of the health-related intervention program for unemployed people.*

*Amount of training*

Research on the dose-response effect in psychotherapy has identified a curvilinear association between the number of therapeutic sessions and the treatment outcome [52]. Since most health interventions for unemployed people are relatively short (i.e., they happen before the curve flattens), we expect to find a linear positive association between the amount of the intervention (measured in total hours of training and in length of intervention in weeks) and its effect on health.

**H9:**  *The amount of the intervention participants received (measured in (a) hours of participation, and (b) weeks of training) moderates the effectiveness of health-related interventions for unemployed people with stronger effects for large amounts compared to smaller amounts.*

#### 1.2.2. Sociodemographic Determinants of Intervention Success

The negative effect of unemployment on health varies with gender, age, and occupation. Meta-analyses on mental health have demonstrated that the difference between unemployed people and employed people is larger for men than for women [5]. It is also larger for workers from blue-collar jobs compared to employees from white-collar jobs [5]. Furthermore, the psychological well-being of youths appears to be more severely impaired by unemployment than that of adults [3]. Finally, while the association between unemployment duration and mental health is curvilinear, it can generally be stated that long-term unemployed people report worse health than short-term unemployed people [3,5]. Assuming that people who suffer more from unemployment have a larger potential for health improvement than people whose health is already good, we would expect gender, age, social class (measured as level of education), and unemployment duration to moderate the effectiveness of interventions for unemployed people.

**H10:**  *Gender moderates the effectiveness of health-related interventions for unemployed people with stronger effects for men compared to women.*

**H11:**  *Age moderates the effectiveness of health-related interventions for unemployed people with stronger effects for youths compared to adults. *

**H12:**  *Level of education moderates the effectiveness of health-related interventions for unemployed people with stronger effects for individuals from a lower education compared to individuals from a higher education.*

**H13:**  *Unemployment duration moderates the effectiveness of health-related interventions for unemployed people with stronger effects for long-term unemployed people compared to short-term unemployed people.*

### 1.3. Sensitivity Analysis 

The design of extant primary studies scrutinizing the effectiveness of health interventions for unemployed people varies considerably with regard to important characteristics. In order to test whether such variations in study design had an influence on their results, we also conducted moderator tests for the following variables: (1) the explicitly stated goals of the intervention; (2) whether there were signs that the participation in the intervention was not completely voluntary; (3) whether the sample consisted of people with preexisting health conditions; (4) whether the control group received some form of alternative treatment; (5) whether participants were assigned to treatment conditions via randomization procedures. No explicit hypotheses were formulated because we assumed that the effectiveness of the interventions would be robust across variations in study design. We also conducted Egger’s test [53] and the trim-and-fill procedure [54] in order to check for a possible publication bias. 

## 2. Materials and Methods

### 2.1. Search for Primary Studies 

In order to find eligible studies, a search of literature databases was conducted. Given that the topic of the meta-analysis is located at the intersection of different academic disciplines, we used the PubMed, Scopus, and PsycINFO (APA) databases in order to cover medical sciences, social sciences, and psychology. The searches were conducted in December 2021.

The search string consisted of three groups of terms, one including words describing the intended employment status of the sample (e.g., “unemployed” or “job loss”); the second group of search terms consisted of words specifying interventions in general (e.g., “intervention” or “training”) and more specific interventions such as “stress management” or “tobacco cessation”. The third group of terms consisted of words describing various health-related outcome variables such as “health” or “distress” or “depression” or “physical symptoms”, etc. The three groups of search terms were connected with Boolean operators: Within the three groups with an “OR”, and between the three groups with an “AND”. As a result, an abstract had to include at least one term from each of the three groups in order to be counted as a hit (the search strings can be found in the Supplementary Online Material SOM-A). Furthermore, we restricted the search to human subjects and papers written in English or German. If applicable to the database, certain document types that were of low interest for our search were excluded (e.g., tutorials, autobiographies, etc.). 

**Figure 1:**
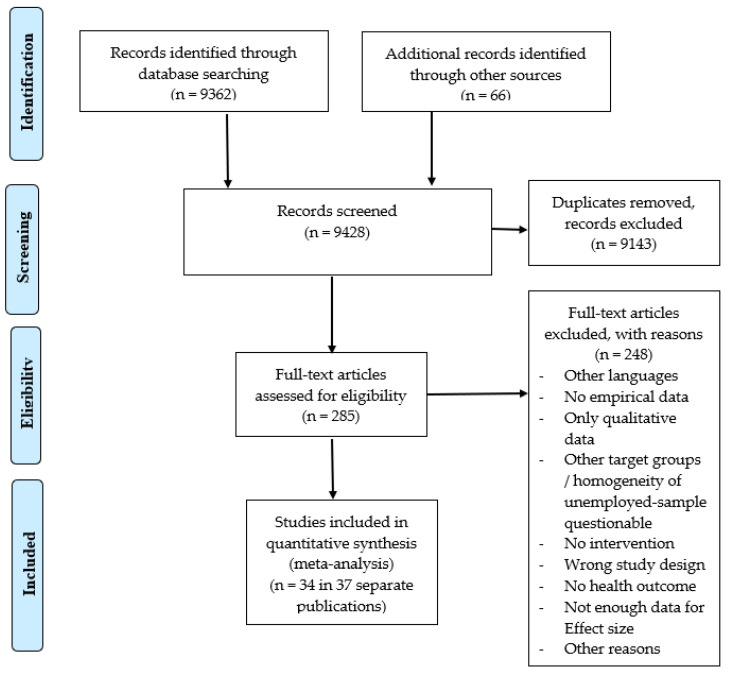
Flow-diagram for literature search and screening process.

These database searches produced altogether 9362 records. Furthermore, 66 additional records were identified through Google Scholar and other sources. Titles and abstracts of these records were screened for eligibility by a research assistant. This screening resulted in 285 records that were categorized as “possibly eligible” (after the exclusion of double entrances). For these records, full-text versions were retrieved, and each full-text was checked for eligibility in the meta-analyses by one of the authors. If one author felt unsure concerning the eligibility of the report, he consulted the other author, and a consensual decision was reached. As a result, 34 individual studies with 36 stochastically independent samples described in 36 different articles and other reports met the inclusion criteria and were included in the meta-analysis (for the various reasons for the inclusion/exclusion of primary studies, see Figure 1).

### 2.2. Inclusion and Exclusion Criteria 

#### 2.2.1. Inclusion Criteria

The goal of the present investigation was to meta-analytically integrate studies testing the effectiveness of interventions aimed at improving unemployed people’s health or health behavior. We therefore formulated the following inclusion criteria: 

(1) The study tested the effectiveness of an intervention, i.e., some sort of treatment that was different from the standard procedures unemployed people receive in a given country. 

(2) The intervention was health-oriented, i.e., aimed at improving the mental or physical health of participants, either in general or with respect to specific aspects of health, or it aimed at improving their health-related behavior. Studies with mixed targets (e.g., fostering reemployment as well as health improvement) were also included. 

(3) The study design involved at least two measurement times (pre-post) and a comparison group that did not receive the intervention (or received it later, after having served as a comparison group). 

(4) The study measured and reported quantitative health-related data. 

(5) The sample of participants consisted of unemployed people, i.e., people who were out of paid work but looking for paid work and who were available for the labor market [55]. 

#### 2.2.2. Exclusion Criteria

A primary study fitting the inclusion criteria was nevertheless excluded from further analyses if: 

(1) Not enough data were reported to allow the computation of an effect size. 

(2) The study report was not written in either English or German. 

(3) In addition, a few studies that matched the originally formulated inclusion criteria were not included because their outcomes were too rarely scrutinized (three or fewer studies) to justify a meta-analytic integration, e.g., smoking cessation or weight reduction.

### 2.3. Outcome Variables 

We included primary studies testing the effectiveness of interventions on three different types of outcome variables: (1) mental health/psychological well-being, (2) physical health, and (3) health behavior. 

*Mental health:* The following variables were accepted as relevant measures of mental health for the present investigation: (1) mixed mental health symptoms; (2) depression; (3) anxiety; (4) self-esteem; and (5) subjective well-being (life satisfaction/positive affect). These variables have been shown to be highly intercorrelated among unemployed people and have high loadings on a common higher-order factor of mental health [5]. 

*Physical health:* We used the following measures of self-reported physical health for the present investigation: (1) general health (self-report measures describing an individual’s health situation in a general, overarching manner), and (2) self-reported physical symptoms (comprising summary scores of lists of different physical symptoms a person might be ailing from). 

*Health behavior:* In addition to measures of mental and physical health, we also included physical activity, which does not represent a direct measure of unemployed people’s health but has a high likelihood of influencing their health. (For other measures of health behavior that we intended to meta-analyze, in particular alcohol and tobacco consumption or nutritional behavior, we could not identify enough primary studies to conduct a meaningful meta-analysis.) 

### 2.4. Coding of Relevant Data 

For the purpose of describing the typical sample used in the intervention studies and for moderator analyses, the following demographic variables were coded for each sample: (1) Gender (percentage of females in sample), (2) age (average age of sample), (3) education (years of formal education), (4) duration of unemployment (average duration in months). (We also tried to code the proportion of blue-collar workers in each sample. However, only very few studies provided detailed information on the former occupations of their participants. The same was true for whether or not participants had an intimate partner, which was also reported in only very few studies).

Furthermore, the following variables concerning the characteristics of the interventions were coded: (1) a series of variables denoting whether each of the intervention techniques specified in hypotheses 5 to 12 (e.g., cognitive-behavioral techniques, motivational interview, relaxation, etc.) was used or was not used in the study; (2) variables describing formal aspects of the intervention. These were: (a) the average number of participants per training/counseling group; (b) and (c) the amount/dose of training the average participant received [measured either as the length of the intervention in weeks from the first to the last session or in total hours each participant was in direct contact with the trainer(s) or counselor(s)]. 

Finally, we also coded the following design aspects of each study: (1) the year of the publication; (2) the language of the study report (English or German); (3) whether health improvement was the only goal of the intervention program (vs. other goals being incorporated, e.g., reemployment); (4) whether randomization was used when allocating the participants to the arms of the study; (5) whether the sample of participants suffered from pre-existing health problems; (6) whether the participation was voluntary or mandatory; (7) whether the control group received any form of treatment or remained untreated. 

All coding was conducted by two research assistants under the close supervision of the first author. In the beginning, the research assistants received an introduction to each of the target variables and a list of examples for possible ensuing coding problems, together with instructions on how to solve them. Next, the research assistants independently coded all variables. Then, the coding decisions were compared, and in cases of discrepancies, the erroneous coding was corrected. In cases of uncertainty over the correct decision, the problem was discussed with the first author, and a consensual decision was reached. 

Interrater agreement after the step of independent coding was measured with Cohen’s Kappa for categorical variables and intraclass correlations for continuous variables. For the abovementioned variables, the median Kappa was *K* = 0.72 (min = 0.15, max = 1.00), and the median intraclass correlation was ICC = 0.88 (min = 0.45, max = 1.00). Thus, the resulting coefficients were indicative of a good level of interrater reliability [56,57]. 

### 2.5. Effect Sizes 

Effect sizes were computed as Cohen’s *d*, i.e., the standardized mean difference between the intervention group and comparison group. The standardized mean difference was computed with the respective formula reported in [58] and then corrected for small-sample bias as suggested by [59]. We computed an effect size at three measurement points, i.e., pre-intervention and post-intervention, and at a later follow-up, if such a follow-up measurement had been conducted.

In most studies, the necessary means and standard deviations were directly reported and could be used for the computation of the effect sizes. In two studies, standard deviations for the control groups were (partly) not reported. We imputed the standard deviations from the intervention group in these cases. In one study, standard deviations were only reported for the first measurement point. We imputed these standard deviations for the second measurement point. In the case of one study where standard deviations were completely missing [60], we imputed the standard deviations from a similar study using identical measurement instruments with unemployed people from the same country undergoing a similar intervention [13]. Such imputations of appropriate standard deviations are a legitimate method of overcoming the common meta-analytic problem that SDs are sometimes missing in primary studies [61,62]. 

Some studies reported other coefficients, such as odds ratios or the results of Mann–Whitney U tests, that allowed the computation of an effect size. These coefficients were converted using the *psychometrica* effect size calculator [63] and a calculator provided by [64]. In a few cases, when the only available information concerning a group comparison was verbal statements about the non-emergence of an effect, we coded a null effect (*d* = 0.00). 

*Situations with more than one treatment or comparison group*

In one study, results for the intervention were reported for three subsamples. Since the groups differed only with regard to geographic location, we computed the combined averages of means and SDs, i.e., we treated the three groups as one large group [62]. In two other studies, two different intervention groups were each compared with their own control group. We kept these sub-studies separate because they differed with regard to important characteristics of the intervention. 

*Multiple outcomes*

In several studies, mental health was represented by more than one measure (e.g., by a scale for depression as well as a scale for anxiety). Since we assumed the different indicators of mental health analyzed here to be equivalent, averaging the effect sizes was an appropriate way of handling this kind of multiplicity [65]. Combining the multiple effect sizes in such cases is expected to result in a better estimate of the true effect than a single effect size [66]. The correct variance of the resulting composite effect sizes was computed with the method described in [67]. This method requires knowledge of the correlation between the respective health outcomes. When these correlations were not directly reported in the primary study, we imputed them from a table of meta-analytically derived average intercorrelations between typical mental health variables such as anxiety and depression. These intercorrelations were based on a large sample of studies on the mental health of unemployed people [5]. Thus, the fit to the samples in the present meta-analysis can be assumed to be very high. 

Multiplicity of outcome measures was a frequent phenomenon only in the domain of mental health, while it occurred only twice in the domain of physical health. Since no intercorrelations were reported in the respective studies on physical health and health behavior, we assumed a correlation of *r* = 1.00. This is the most conservative solution because it leads to large variances for the respective studies and thus small weights in the meta-analysis [67]. 

### 2.6. Meta-Analytic Model

We used a random-effects model with restricted maximum-likelihood estimation to synthesize the results because intervention methods, as well as study designs, differed considerably between the available primary studies. Furthermore, we intended to generate findings that could be generalized to the whole research field and allow predictions for future intervention studies for unemployed people [68]. Computations were done with SPSS, version 28.0.0.0. Heterogeneity was assessed via *I*^2^ and prediction intervals. Moderator tests were conducted with meta-regression (for continuous moderators) and subgroup analysis (for categorical moderators). Because of the small number of existing intervention studies for unemployed people and the resulting low power of our moderator tests, we also—very cautiously—interpreted marginally significant results at the level of *p* < 10.

## 3. Results

### 3.1. Characteristics of the Included Primary Studies

The large majority of the 34 intervention studies meta-analyzed here were conducted in Europe (52.8%), while 22.2% were conducted in Australia and 16.7% in North America (see Supplementary Online Material B for a list of the 36 primary studies). Only two studies (5.6%) were conducted on other continents, i.e., in South Korea and South Africa. The year of publication varied between 1989 and 2020, with three-quarters of the studies having been published in 2000 or later. 

The average number of unemployed people per intervention group was Md = 9.0 participants per group. The duration of the typical intervention program was Md = 3.0 weeks, with a range from min = 1 day to max = 26.0 weeks. With regard to the amount of treatment participants received, studies typically had Md = 20 h of contact time, but with a large range from min = 1 h to max = 220 h. The most frequent types of intervention were cognitive-behavioral methods (41.7%) and training in job-search techniques (41.7%), followed by non-cognitive stress-management techniques (33.3%), relaxation techniques (27.8%), and methods aiming at an increase in social support (25.0%). All other methods were employed in less than one-fifth of studies.

With regard to demographic characteristics, slightly more than half (Md = 54.0%) of the participants in a typical study were women (ranging from Min = 8.9% to Max = 100%), and the average age was Md = 41.2 years (ranging from Min = 19.0 to Max = 54.9). In order to measure social class, we coded the average years of formal education, which was Md = 12.0 years with a range from Min = 9.7 years to Max = 14.0 years. Furthermore, in the typical study, about a third of the participants were married or in a lasting relationship (Md = 33.8%; min = 13.1%, max = 50.0%). 

The average length of joblessness in the sample of unemployed participants was often very long, with a minimum of Min = 1.0 months, a maximum of Max = 132.0 months, and a mean of Md = 36.0 months. 

Randomization was conducted in 61.1% of the primary studies. 61.1% of the studies used a control group with no or only minimal treatment (no training at all, waiting list, or booklet); 30.6% of the control groups received some form of training course that can be regarded as a part of the typical treatment unemployed people receive in most countries, particularly job search training; and 8.3% of the control groups received some form of alternative treatment that was specifically designed for the respective study. Furthermore, 22.2% of the samples were characterized by some form of pre-existing health impairment, and in 13.9% of the studies, we identified signs that participation in the intervention was not voluntary, e.g., because it was imposed by a job agency.

### 3.2. Effects of Interventions on Mental Health, Physical Health, and Health Behavior

For the health measurements taken before any intervention happened, no significant effect sizes could be found (see Table 1, Table 2 and Table 3 first line). The effect sizes were very small and not significant for mental health, *d* = −0.06; 95% CI [−0.16, 0.04], as well as physical health, *d* = 0.07; 95% CI [−0.00, 0.13], and health behavior/physical activity, *d* = −0.02; 95% CI [−0.24, 0.20]. Thus, it is unlikely that health-related biases have influenced the allocation of participants to the intervention and control conditions in the primary studies meta-analyzed here. 

At the second measurement point, i.e., after the intervention, significant group differences were found for mental health and physical activity (see Table 1 and Table 3, second line): Members of the treatment groups showed significantly better mental health than members of the comparison groups, *d* = 0.22; 95% CI [0.08, 0.36], and also showed significantly better health behavior (physical activity), *d* = 0.30; 95% CI [0.13, 0.47]. For mental health, the effect was of small size. For health behavior, it was of small-to-medium size. For physical health, a small effect was found that was marginally (*p* = 0.10) significant, *d* = 0.09; 95% CI [−0.02, 0.20].

At the follow-up (see Table 1, Table 2 and Table 3, third line), the average effect size for mental health was smaller than directly after the intervention, but still significant, *d* = 0.11; 95% CI [0.07, 0.16], indicating that the participants of the treatment groups still had better mental health than the members of the comparison groups. For physical health, the effect at follow-up was very small and not significant, *d* = 0.03; 95% CI [−0.12, 0.19]. There were not enough data on health behavior (physical activity) available to conduct a meta-analysis at follow-up. In summary, hypotheses 1a and 1c were supported by the meta-analytic results: Health-oriented interventions for unemployed people have a positive effect on mental health and physical activity. Regarding H1b, only weak evidence supporting a positive effect on physical health was found. 

### 3.3. Moderator Analyses—Type of Intervention

Moderator tests concerning the type of intervention revealed no significant results for mental health (see Supplementary Online Material, Table S1). (We report only results with at least four primary studies in each subgroup for categorical moderators in order to ascertain a sufficient stability of findings. Moderation analyses for continuous moderators are only reported when they are based on at least ten primary studies (see [69])). 

The average effect size was larger for studies using cognitive-behavioral techniques than for studies not using such techniques, but the difference was not significant, neither after the intervention (*d* = 0.29; 95% CI [0.05, 0.53] vs. *d* = 0.17; 95% CI [−0.01, 0.34], (*Q*_b_ = 0.64, *p* = 0.43), nor at follow up (*d* = 0.18; 95% CI [0.05, 0.30] vs. *d* = 0.10; 95% CI [0.05, 0.16], (*Q*_b_ = 1.08, *p* = 0.30). Thus, studies using CBT appeared to be more effective at first glance, as expected in H2, but this result was not supported by significance testing. 

For all other methods (non-cognitive stress management techniques, relaxation techniques, strengthening of social support, job search training, physical exercise, and health counseling), there was also no empirical support for their effectiveness being different from the typical effectiveness of methods used in intervention studies for unemployed people, i.e., none of the moderator tests was significant. Thus, while the typical intervention study had a significant positive effect on mental health (see Section 3.2), we were not able to identify a specific type of intervention that was clearly superior or inferior compared to the others. Effectivity appears to be similar for all the different types of intervention for unemployed people analyzed here. 

With regard to physical health (see Table S2), we identified a significant moderator effect for the inclusion of job-search training methods into the intervention program (*Q*_b_ = 8.59, *p* = 0.003). Studies using job-search training had *smaller* effect sizes (*d* = −0.11; 95% CI [−0.26, 0.05] than studies not using this method, *d* = 0.17; 95% CI [0.07, 0.27]. Indeed, only for the group of studies abstaining from using job search training did we find a significantly positive effect of the intervention programs on physical health.

We found no significant moderating effects for the use of the other intervention methods analyzed here (CBT, non-CBT stress management, relaxation, physical exercise, health counseling). 

It might be noted, however, that the average effect for studies using cognitive-behavioral techniques was significant, *d* = 0.15; 95% CI [0.04, 0.27], as was the average effect for studies using relaxation techniques, *d* = 0.19; 95% CI [0.04, 0.33], physical exercise, *d* = 0.20; 95% CI [0.05, 0.35], and health counseling, *d* = 0.18; 95% CI [0.02, 0.35], while this was not the case for studies that did abstain from using the respective methods. 

In summary, no clear evidence supporting any of the hypotheses H1 to H7 could be identified. Neither for mental nor physical health. A significant moderator effect for the use of job search training methods was found, though, showing that they reduced intervention effectiveness for physical health. For health behavior, moderator tests were not possible due to the very small number of available primary studies. 

### 3.4. Moderator Analyses—Formal Aspects of the Intervention

No significant moderating effects were found for the size of the training group, neither for mental health nor for physical health, rejecting H8 (see Tables S3 and S4). 

The intensity of the interventions, measured as hours of contact time with trainers, also had no significant influence on the effect size for mental health. Yet, for physical health, we found a significant moderating effect when one study with an unusually high amount of contact time was excluded and intensity was thus limited to max. 40 h. More contact time was associated with larger effect sizes, i.e., a better effectiveness of the intervention (*β* = 0.011; *p* = 0.014). 

Moderator tests for the duration of the intervention were initially also not significant. However, when four studies with unusually long durations were excluded (effectively restricting the maximum intervention time to nine weeks), a significant positive moderating effect on mental health could be identified (*β* = 0.061; *p* = 0.041). Thus, within the upper limit of about two months, interventions with a longer duration were more effective for mental health than interventions with a shorter duration. For physical health, no significant moderating effect for the length of the study was found. We conclude that hypothesis 9 was partly supported. 

### 3.5. Moderator Analyses—Demographic Characteristics

Contrary to our expectations, all moderator tests for the demographic characteristics of the samples included in the meta-analysis were insignificant (see Tables S5 and S6). Neither age, gender, nor social class measures such as years of formal education had an influence on the effectiveness of intervention programs for mental or physical health. The only (partial) exception was the analysis for unemployed duration, which revealed a marginally significant positive effect on mental health, meaning the program effectiveness may have been slightly larger when the study participants had a high average duration of unemployment. Thus, H10–H12 were not endorsed, and H13 received weak empirical support. 

### 3.6. Sensitivity Analysis 

The effectiveness of the interventions meta-analyzed here varied greatly, as demonstrated by the large prediction intervals accompanying the average effect sizes. These intervals all included the null, indicating a large variability between studies and the impossibility of predicting whether a specific new study that tries to improve unemployed people’s health will be successful or not. 

Furthermore, we analyzed whether variations in the design and the formal aspects of the studies influenced their outcomes (see Tables S7 and S8). With one exception, this was not the case. Neither the language of the study report (mental health: *Q*_b_ = 0.47, *p* = 0.493/physical health: *Q*_b_ = 1.21, *p* = 0.272), nor the number of goals of the intervention (mental health: *Q*_b_ = 2.30, *p* = 0.129/physical health: *Q*_b_ = 1.20, *p* = 0.274), nor whether the sample consisted of people with pre-existing health impairments or not (mental health: *Q*_b_ = 0.08, *p* = 0.778 / physical health: *Q*_b_ = 0.44, *p* = 0.506), nor the use of randomization (mental health: *Q*_b_ = 1.59, *p* = 0.208 / physical health: *Q*_b_ = 1.78, *p* = 0.183), nor whether the control group received some treatment or not (mental health: *Q*_b_ = 2.48, *p* = 0.115 / physical health: *Q*_b_ = 1.21, *p* = 0.272) had a significant influence on the effectiveness of the intervention programs. 

The only exception was the voluntariness of participation in the intervention. For this variable, a significant moderating effect was revealed for mental health (*Q*_b_ = 6.04, *p* = 0.014). Interventions had a significant positive effect on mental health only when unemployed people participated out of their own free will, *d* = 0.29; 95% CI [0.15, 0.43]. In contrast, when we found signs of limited voluntariness, for example, because a job center made participation in the course mandatory for its unemployed clients, the interventions did not show any positive effectiveness, *d* = −0.14; 95% CI [−0.45, 0.17]. 

Finally, publication bias did not emerge as a relevant threat to the validity of the meta-analytic outcomes (see notes for Table 1, Table 2 and Table 3). Egger’s test was significant at follow-up for the analysis regarding mental health, but the imputation of three studies during the trim-and-fill procedure only led to an extremely small change in the average effect size. Thus, the asymmetry indicated by Egger’s test appeared to be inconsequential for this analysis. 

For the other analyses testing the effectiveness of health-related interventions, Egger’s test was not significant, and the trim-and-fill method usually indicated no or very few missing studies. In no case would the imputations suggested by this method in order to achieve a symmetric distribution of studies have led to a noteworthy change in the meta-analytic mean effect size.

## 4. Discussion

The present meta-analyses provided evidence that, on average, interventions aiming at improving unemployed people’s health are successful, with participants of intervention groups reporting better health after the intervention than participants of control groups. This is particularly true for mental health and physical activity. At follow-up, the positive effect on mental health was still significant, demonstrating that the interventions do not just lead to a fleeting boost in well-being that quickly fades but that the effect has a certain stability over several months. For physical health, the overall analysis was not significant, with only a weak trend pointing towards effectiveness. However, this finding was qualified by a significant moderator effect for the inclusion of job search training into the intervention program. When job search training was included, no positive effect emerged. However, when job search training was not included, a significant positive effect of interventions on physical health could be identified. 

The intervention effects were generally weak. Yet, given the relatively limited average duration (3 weeks) and intensity (20 h) of the typical intervention for unemployed people, larger effect sizes could probably not be expected. From this pattern of results, refresher courses appear to be advisable in order to achieve a sustained positive health effect over longer time spans. Courses should not be mandatory, however.

The results found here are in good agreement with the conclusions of earlier reviews, which concluded that at least some interventions for unemployed people were able to improve their health [26,28] and that positive effects were more frequent for mental health compared to physical health [26]. The positive influence of voluntariness on participation has also been observed before [26], although these reviews did not use quantitative methods of research integration. The only pertinent review that used meta-analytic techniques [70] was primarily concerned with reemployment as an outcome, not with effects on health. It reported small and non-significant effect sizes for intervention effectiveness on mental health and general health. Yet, since only two studies were included in those computations, we believe the present results to be based on higher test power and to better represent the research field. 

Regarding the question of which intervention techniques are more or less successful than others, the results were equivocal. No moderator test was significant, clearly showing whether or not it is advisable to include a specific technique in a program or not. This should not be interpreted as indicating that none of the intervention methods were effective, however. The reason is that the moderator tests compared the effect sizes of studies using a specific technique not with studies using no technique at all, but with studies using other—possibly helpful—techniques. Our method of moderator testing implies that if all intervention methods were equally effective, no moderator test would become significant, as was the case here.

Nevertheless, the results allow only tentative suggestions about the selection of intervention methods for unemployed people. For mental health, the results show that interventions were often successful when they included cognitive-behavioral methods. This finding is also in good agreement with earlier reviews [26,28]. For other methods, the effect sizes for studies using them tended to be smaller and were sometimes insignificant. A positive message is that the inclusion of job search trainings (which are often used in combination with more health-oriented techniques) appears not to have a deleterious influence on the effectiveness of health-related intervention programs for unemployed people, at least with regard to mental health. 

With regard to self-reported physical health, interventions including CBT methods, relaxation techniques, physical exercise, and health counseling were successful, insofar as the average effect sizes for studies using one of these methods were positive and significant, although the effects were usually small. In contrast, the use of non-CBT-based stress management techniques did not lead to a significantly positive average effect on physical health. An explanation might be that most interventions in this heterogenous category have rarely been used with unemployed people so far, inhibiting researchers from learning from the experiences of earlier trials with this specific social group.

Furthermore, for physical health—in contrast to the results for mental health—interventions were only significantly effective when they abstained from including elements of job search training into their programs. Adding additional goals to the main goal of health improvement, particularly the goal to find a new job, might have indirect positive effects on mental health (for example, via a boost in hope and self-efficacy) that possibly neutralize the problem of dividing time and resources between two or more different goals. For physical health, however, an improvement might require the full concentration of all available resources on this specific goal if an intervention for unemployed people is meant to be successful. 

With regard to the formal characteristics of the interventions, the moderator analyses showed that within the upper limit of about 9 weeks, longer durations of the programs tend to be more successful than shorter trainings. In a similar vein, within the upper limit of 40 h of contact time with the trainers, a higher intensity of training tends to be more promising than a lower intensity. 

The expected pattern for demographic characteristics with social groups who suffer more from unemployment profiting more from health-oriented interventions was only identified as a weak trend for unemployment duration, with studies with participants who were longer unemployed reporting a better effectiveness than studies with participants whose unemployment was relatively short. This finding gives a tentative first insight into what might be promising target groups for future interventions. 

### Limitations

The methodological quality of the primary studies that were integrated here was rather mixed. For example, more than a third of the studies did not apply randomization procedures. However, despite this partial lack of randomization, there were no health-related differences between intervention groups and control groups at the first measurement point, i.e., before the start of the intervention. This implicates that self-selection or intentional assignment of specific participants to treatment conditions by job-agency officials did not lead to biased group compositions at the beginning of the interventions. 

Another possible problem was the treatment of the control groups. Several studies compared their intervention not with a non-treatment condition but with some form of alternative treatment, ranging from standard training courses for unemployed people to camp-like outdoor courses for youngsters or introductory first-aid courses. Yet, s moderator test for the treatment of the control group could not identify a significant difference in effectiveness, with control groups without alternative treatment having similar effects to control groups with some form of alternative treatment. 

Differential selection into the intervention could theoretically also have affected the outcomes of the meta-analysis, at least in those studies where no randomization was conducted. For example, researchers or employment agency staff might have been biased toward including people with worse health in the intervention group because they perceived the need for help to be particularly strong in these cases. However, a moderator test found no differences in intervention effectiveness between samples with and without health limitations (see Supplementary Materials). It is therefore unlikely, in our eyes, that health-related selection into the intervention (if it existed) could have strongly influenced the results of the meta-analysis. 

Furthermore, participation rates regularly dropped from the beginning to the end of the interventions. Regrettably, the primary studies usually did not report health-related data, allowing a comparison of the drop-outs with those participants who continued with their participation until the end of the intervention. As a consequence, we do not have data enabling us to test the existence of health-related differential dropout throughout the course of the interventions. We do believe the resulting threat to the validity of our findings to be limited, however, because there is no straightforward reason why sudden health deteriorations—precluding further participation—should have affected more people in either the intervention group or the control group. 

Furthermore, the present analysis was restricted to studies in English and German. However, since we did not find differences between studies published in English versus German, we do not see a reason why studies published in other languages should deviate from this pattern. Thus, a risk to the validity of our conclusions is unlikely in our eyes. Finally, publication bias appears to be a limited threat to the validity of the results presented here because tests checking the symmetry of the distribution of effect sizes found only very weak signs of asymmetry, i.e., a low likelihood of unsuccessful studies being routinely repressed during the publication process. 

## 5. Conclusions

The present meta-analysis confirms that it is possible to improve unemployed people’s health with relatively limited expenditures, i.e., by using relatively short group-based programs with limited contact times. Furthermore, health impairments have been shown to have a negative influence on labor market success [5]. Thus, health-oriented interventions for unemployed people might do even more than improve their health. In the medium- or long-term perspective, they may also increase participants’ chances for re-employment. In line with that assumption, a recent review on this topic found a weak but positive effect of health-improving interventions for unemployed job seekers on the likelihood of obtaining re-employment [70]. 

Since unemployment is a problem that affects a significant part of the population—millions of people—such interventions could have a strong impact on public health if they were applied on a large scale, despite their relatively small effectiveness. Thus, population-based health promotion programs are recommended because even measures with small effect sizes can actually improve the health of a large group of unemployed people (“prevention paradox”).

## Figures and Tables

**Table 1:** Intervention Effect on Mental Health.

Time	*N*	*k*	*d*	*SE*	*p*	95% CI	*I* ^2^	95% PI
Pre-intervention	6592	31	−0.06	0.0495	0.225	[−0.16, 0.04]	67.4%	[−0.48, 0.36]
Post-intervention	6948	32	0.22	0.0717	0.003	[0.08, 0.36]	88.0%	[−0.51, 0.94]
Follow-up	3414	16	0.11	0.0247	<0.001	[0.07, 0.16]	0.0%	[0.06, 0.17]

Note: *k* = number of effect sizes; *N* = sample size; *d* = average meta-analytic effect size; *SE* = standard error for *d*; *p* = significance level for *d*; 95% CI = 95% confidence interval for *d*; *I*^2^ = proportion of unexplained heterogeneity; PI = 95% prediction interval for *d*. *Pre-intervention:* Egger’s test not significant (*p* = 0.767); imputation of two studies during trim-and-fill procedure led to a very small decrease in the effect size: *d* = −0.05; 95% CI [−0.16, 0.04]. *Post-intervention:* Egger’s test not significant (*p* = 0.993); trim-and-fill analysis led to no imputations. *Follow-up:* Egger’s test significant (intercept = 0.086, *SE* = 0.0357, *p* = 0.031); three studies were imputed in trim-and-fill procedure, leading to a very small decrease in the effect size: *d* = 0.11; 95% CI [0.06, 0.17].

**Table 2:** Intervention Effect on Physical Health.

Time	*N*	*k*	*d*	*SE*	*p*	95% CI	*I* ^2^	95% PI
Pre-intervention	3658	16	0.07	0.0348	0.061	[−0.00, 0.13]	3.6%	[−0.03, 0.16]
Post-intervention	2599	16	0.09	0.0552	0.100	[−0.02, 0.20]	42.3%	[−0.22, 0.40]
Follow-up	771	5	0.03	0.0776	0.663	[−0.12, 0.19]	0.0%	[−0.21, 0.28]

*Note*: *k* = number of effect sizes; *N* = sample size; *d* = average meta-analytic effect size; *SE* = standard error for *d*; *p* = significance level for *d*; 95% CI = 95% confidence interval for *d*; *I*^2^ = proportion of unexplained heterogeneity; PI = 95% prediction interval for *d*. *Pre-intervention:* Egger’s test significant at T1 (intercept = 0.186, *SE* = 0.0815, *p* = 0.039); five studies were imputed in trim-and-fill procedure, leading to an increase in the effect size: *d* = 0.10; 95% CI [0.02, 0.17]. *Post-intervention:* Egger’s test not significant (*p* = 0.642); imputation of one study during trim-and-fill procedure led to a very small decrease in the effect size: *d* = 0.08; 95% CI [−0.03, 0.19]. *Follow-up:* Egger’s test not significant (*p* = 0.584); imputation of two studies during trim-and-fill procedure led to a small increase in the effect size: *d* = 0.08; 95% CI [−0.05, 0.21].

**Table 3:** Intervention Effect on Physical Activity.

Time	*N*	*k*	*d*	*SE*	*p*	95% CI	*I* ^2^	95% PI
Pre-intervention	812	5	−0.02	0.1128	0.876	[−0.24, 0.20]	39,8%	[−0.62, 0.59]
Post-intervention	730	5	0.30	0.0869	<0.001	[0.13, 0.47]	11,9%	[−0.06, 0.65]

*Notes*: *k* = number of effect sizes; *N* = sample size; *d* = average meta-analytic effect size; *SE* = standard error for *d*; *p* = significance level for *d*; 95% CI = 95% confidence interval for *d*; *I*^2^ = proportion of unexplained heterogeneity; PI = 95% prediction interval for *d*. *Pre-intervention:* Egger’s test not significant (*p* = 0.839); two studies were imputed in trim-and-fill procedure, increasing effect size to: *d* = 0.11; 95% CI [−0.14, 0.35]. *Post-intervention:* Egger’s test not significant (*p* = 0.233); no study imputed in trim-and-fill procedure.

## Data Availability

A list of included primary studies, a table with codings of study characteristics, and forest plots depicting each primary study’s effect size and confidence interval are provided in the online Supplementary Materials [73,74,75]. Further material is available from the corresponding author upon reasonable request.

## Supplementary Materials

The following supporting information can be downloaded at: https://www.mdpi.com/article/10.3390/ijerph20116028/s1. (1) Search strings for the literature search; (2) Tables documenting the results of the moderator analyses; (3) An alphabetic list of included primary studies [11,12,14,15,16,17,18,19,32,33,34,60,71,72,73,74,75,76,77,78,79,80,81,82,83,84,85,86,87,88,89,90,91,92,93,94,95]; (4) Forest plots depicting each primary study’s effect size and confidence interval; (5) Tables with the codings of study characteristics.
