# Supplementary material for: The Effectiveness of Health-Oriented Interventions and Health Promotion for Unemployed People—A Meta-Analysis
Source: Int J Environ Res Public Health. 2023 Jun 1;20(11):6028. doi: 10.3390/ijerph20116028 (PMC10252930; doi:10.3390/ijerph20116028)
Supplement: Supplementary file 1 [file ijerph-20-06028-s001.zip › ijerph-2165440-supplementary.pdf]

The Effectiveness of Health-Oriented Interventions and Health Promotion for Unemployed People – a Meta-Analysis

Karsten Ingmar Paul and Alfons Hollederer

SUPPLEMENTARY ONLINE MATERIAL - A: Search strings

Search strings

For the PubMed database:

(unemploy* [TIAB] OR jobseeker [TIAB] OR "job loss"[TIAB] OR reemploy*[TIAB]) AND (program [TIAB] OR intervention[TIAB] OR "health promotion"[TIAB] OR "primary prevention"[TIAB] OR counsel*[TIAB] OR training[TIAB] OR "physical activity"[TIAB] OR "smoking prevention"[TIAB] OR "tobacco cessation"[TIAB] OR exercise[TIAB] OR diet[TIAB] OR "stress management"[TIAB] OR "health literacy"[TIAB] OR "body weight change"[TIAB] OR "suicide prevention"[TIAB] OR "behavior coping"[TIAB]) AND (health [TIAB] OR distress[TIAB] OR depression[TIAB] OR well-being[TIAB] OR anxiety[TIAB] OR psychosomatic[TIAB] OR somatization[TIAB] OR self-esteem[TIAB] OR "sense of coherence"[TIAB] OR "physical symptoms"[TIAB] OR alcohol[TIAB] OR smoking [TIAB] OR sport [TIAB] OR "physical activity" [TIAB])

For the PsycInfo database:

(AB (unemploy* OR jobseeker OR "job loss" OR reemploy*) OR TI (unemploy* OR jobseeker OR "job loss" OR reemploy*)) AND (AB (program OR intervention OR "health promotion" OR "primary prevention" OR counsel* OR training OR "physical activity" OR "smoking prevention" OR "tobacco cessation" OR exercise OR diet OR "stress management" OR "health literacy" OR "body weight change" OR "suicide prevention" OR "behavior coping") OR TI (program OR intervention OR "health promotion" OR "primary prevention" OR counsel* OR training OR "physical activity" OR "smoking prevention" OR "tobacco cessation" OR exercise OR diet OR "stress management" OR "health literacy" OR "body weight change" OR "suicide prevention" OR "behavior coping")) AND (AB (health OR distress OR depression OR well-being OR anxiety OR psychosomatic OR somatization OR self-esteem OR "sense of coherence" OR "physical symptoms" OR alcohol OR smoking OR sport OR "physical activity") OR TI (health OR distress OR depression OR well-being OR anxiety OR psychosomatic OR somatization OR self-esteem OR "sense of coherence" OR "physical symptoms" OR alcohol OR smoking OR sport OR "physical activity"))

For the Scopus database:

(unemploy* OR jobseeker OR {job loss} OR reemploy*) AND (program OR intervention OR {health promotion} OR {primary prevention} OR counsel* OR training OR {physical activity} OR {smoking prevention} OR {tobacco cessation} OR exercise OR diet OR {stress management} OR {health literacy} OR {body weight change} OR {suicide prevention} OR {behavior coping}) AND (health OR distress OR depression OR well-being OR anxiety OR psychosomatic OR somatization OR self-esteem OR {sense of coherence} OR {physical symptoms} OR alcohol OR smoking OR sport OR {physical activity})

SUPPLEMENTARY ONLINE MATERIAL - B: Tables documenting the results of moderator tests

Table S1: Moderator tests for intervention effectivity on mental health: Type of intervention

Variable	Time		Q_b	*p*	*k*	*d*	*p*	95% CI
CBT	Post-intervention	Yes	0.64	0.426	14	0.29	0.019	[0.05, 0.53]
		No			18	0.17	0.063	[-0.01, 0.34]
	Follow-up	Yes	1.08	0.298	7	0.18	0.007	[0.05, 0.30]
		No			9	0.10	<0.001	[0.05, 0.16]
Non-CBT Stress Management	Post-intervention	Yes	0.00	0.959	12	0.21	0.052	[-0.00, 0.42]
		No			20	0.22	0.024	[0.03, 0.40]
	Follow-Up	Yes	0.31	0.577	6	0.11	<0.001	[0.05, 0.16]
		No			10	0.14	0.010	[0.03, 0.25]
Relaxation techniques	Post-intervention	Yes	0.54	0.463	10	0.15	0.046	[0.00, 0.30]
		No			22	0.24	0.011	[0.06, 0.43]
	Follow-Up	Yes	0.20	0.658	4	0.15	0.087	[-0.02, 0.32]
		No			12	0.11	<0.001	[0.06, 0.16]
Strengthening social support	Post-intervention	Yes	0.00	0.954	9	0.21	0.044	[0.01, 0.41]
		No			23	0.22	0.021	[0.03, 0.40]
	Follow-up	Yes	0.24	0.624	6	0.11	<0.001	[0.06, 0.16]
		No			10	0.14	0.027	[0.02, 0.27]
Job-search training	Post-intervention	Yes	0.92	0.761	15	0.24	0.015	[0.05, 0.43]
		No			17	0.19	0.074	[-0.02, 0.41]
	Follow-up	Yes	0.42	0.515	9	0.11	<0.001	[0.06, 0.16]
		No			7	0.19	0.035	[0.01, 0.36]
Physical exercise	Post-intervention	Yes	0.58	0.448	4	0.12	0.317	[-0.11, 0.35]
		No			28	0.23	0.005	[0.07, 0.38]
Health counseling	Post-intervention	Yes	1.80	0.180	4	-0.05	0.806	[-0.49, 0.38]
		No			28	0.26	<0.001	[0.12, 0.40]

Note. k = number of effect sizes; *d* = average meta-analytic effect size; CI= 95% confidence interval for *d*; Q_b = heterogeneity between groups; *p* = significance level for *d* or for Q_b;; CBT = cognitive behavioral training.

Table S2: Moderator tests for intervention effectivity on physical health: Type of intervention

Variable	Time		Q_b	*P*	*k*	*d*	*p*	95% CI
CBT	Post-intervention	Yes	1.05	0.306	5	0.15	0.007	[0.04, 0.27]
		No			11	0.05	0.550	[-0.11, 0.21]
Non-CBT stress management	Post-intervention	Yes	1.96	0.161	4	-0.05	0.647	[-0.25, 0.15]
		No			12	0.12	0.053	[-0.00, 0.24]
Relaxation techniques	Post-intervention	Yes	1.62	0.203	4	0.19	0.010	[0.04, 0.33]
		No			12	0.06	0.417	[-0.08, 0.20]
Job-search training	Post-intervention	Yes	8.59	0.003	4	-0.11	0.169	[-0.26, 0.05]
		No			12	0.17	0.001	[0.07, 0.27]
Physical exercise	Post-intervention	Yes	2.11	0.146	4	0.20	0.008	[0.05, 0.35]
		No			12	0.05	0.436	[-0.08, 0.19]
Health counseling	Post-intervention	Yes	2.16	0.141	7	0.18	0.029	[0.02, 0.35]
		No			9	0.02	0.728	[-0.11, 0.16]

Note. *k* = number of effect sizes; *d* = average meta-analytic effect size; CI= 95% confidence interval for *d*; Q_b = heterogeneity between groups; *p* = significance level for *d* or for Q_b;; CBT = cognitive behavioral training.

Table S3: Moderator tests for intervention effectivity on mental health: Formal characteristics of intervention

Variable	*Time*	*k*	*Intercept*	SE_I	β	SE_β	*p*	$I^2_{residual}$
Duration of Intervention (in weeks)	Post-I.	31	0.287	0.0697	-0.006	0.0032	0.078	84.0%
(max 9 weeks)	Post-I.	27	0.085	0.1148	0.061	0.0301	0.041	79.8%
	Fol.-up	16	0.098	0.038	0.008	0.0134	0.561	0.0%
Intensity of intervention (hours of contact)	Post-I.	30	0.190	0.0780	0.002	0.0011	0.139	81.8%
	Fol.-up	16	0.110	0.0319	0.000	0.0008	0.852	0.0%
Size of training groups	Post-I.	21	0.299	0.1921	-0.006	0.0158	0.691	89.7%
	Fol.-up	10	0.154	0.1149	-0.003	0.0088	0.694	0.0%

Note. k = number of effect sizes; SE_I = standard error of intercept; β = regression weight; SE_β = standard error of regression weight; *p* = significance level for beta; $I^2_{residual}$ = heterogeneity between studies remaining unexplained; Post-I. = post intervention; Fol.-up = follow up.

Table S4: Moderator tests for intervention effectivity on physical health – formal characteristics of intervention

Variable	*Time*	*k*	*Intercept*	SE_I	β	$SE_β$	*p*	$I^2_{residual}$
Duration of Intervention (in weeks)	Post-I.	15	0.077	0.0617	-0.001	0.0019	0.623	34.2%
Intensity of intervention (hours of contact)	Post-I.	12	-0.014	0.0782	0.002	0.0018	0.157	31.1%
(max. 40 hours)	Post-I.	11	-0.173	0.1044	0.011	0.0046	0.014	5.1%
Size of training groups	Post-I.	11	-0.059	0.1048	0.004	0.0091	0.695	17.1%

Note. k = number of effect sizes; SE_I = standard error of intercept; β = regression weight; $SE_β$ = standard error of regression weight; *p* = significance level for beta; $I^2_{residual}$ = heterogeneity between studies remaining unexplained; Post-I. = post intervention; Fol.-up = follow up.

Table S5: Moderator tests for intervention effectivity on mental health – demographic characteristics

Variable	*Time*	*k*	*Intercept*	SE_I	β	$SE_β$	*p*	$I^2_{residual}$
Percent. female	Post-I.	32	0.241	0.2170	0.0007	0.0038	0.905	88.0%
	Fol.-up	16	0.174	0.0962	-0.001	0.0015	0.514	0.0%
Age	Post-I.	31	0.291	0.3618	-0.003	0.0089	0.762	86.4%
	Fol.-up	16	0.028	0.2309	0.002	0.0060	0.711	0.0%
Years Education	Post-I.	14	-0.455	0.9979	0.055	0.0830	0.506	89.9%
Unemp. duration	Post-I.	23	0.191	0.1362	-0.002	0.0032	0.581	89.5%
	Fol.-up	14	0.072	0.0339	0.004	0.0023	0.099	0.0%

Note. *k* = number of effect sizes; SE_I = standard error of intercept; β = regression weight; $SE_β$ = standard error of regression weight; *p* = significance level for beta; $I^2_{residual}$ = heterogeneity between studies remaining unexplained; Post-I. = post intervention; Fol.-up = follow up.

Table S6: Moderator tests for intervention effectivity on physical health – demographic characteristics

Variable	*Time*	*k*	*Intercept*	SE_I	β	$SE_β$	*p*	$I^2_{residual}$
Percent. female	Post-I.	14	0.201	0.2152	-0.002	0.0039	0.559	48.3%
Age	Post-I.	16	-0.277	0.4423	0.008	0.0100	0.402	45.5%
Unemp. duration	Post-I.	15	-0.075	0.1281	0.004	0.0028	0.157	41.7%

Note. *k* = number of effect sizes; SE_I = Standard error of intercept; β = regression weight; $SE_β$ = standard error of regression weight; *p* = significance level for beta; $I^2_{residual}$ = heterogeneity between studies remaining unexplained; Post-I. = post intervention.

Table S7: Moderator tests for intervention effectivity on mental health: – characteristics of study design

Variable	Time		Q_b	*p*	*k*	*d*	*p*	95% CI
Language	Post-interven.	English	0.47	0.493	28	0.24	0.001	[0.09, 0.39]
		German			4	0.06	0.794	[-0.42, 0.54]
Study goals	Post-interven.	*Health is…*						
		…only goal	2.30	0.129	11	0.09	0.060	[-0.00, 0.19]
		…one of several goals or secondary goal			21	0.26	0.009	[0.06, 0.46]
Voluntariness of participation	Post-interven.	Likely voluntary	6.04	0.014	27	0.29	<0.001	[0.15, 0.43]
		Involuntary			5	-0.14	0.389	[-0.45, 0.17]
Sample with health impairments	Post-interven.	Yes	0.08	0.778	8	0.27	0.269	[-0.21, 0.74]
		No			24	0.20	<0.001	[0.08, 0.31]
Randomi-zation	Post-interven.	Yes	1.59	0.208	20	0.28	0.001	[0.12, 0.45]
		No			12	0.09	0.497	[-0.16, 0.34]
	Follow-up	Yes	0.38	0.537	10	0.11	<0.001	[0.06, 0.16]
		No			6	0.16	0.044	[0.00, 0.32]
Treatment control group	Post-interven.	No alternative treatment	2.48	0.115	19	0.30	0.004	[0.10, 0.51]
		Alternat. treatment or standard training for unemp. ppl.			13	0.09	0.257	[-0.07, 0.26]

Note. *k* = number of effect sizes; *d* = average meta-analytic effect size; CI= 95% confidence interval for *d*; Q_b = heterogeneity between groups; *p* = significance level for *d* or Q_b.

Table S8: Moderator tests for intervention effectivity on physical health: – characteristics of study design

Variable	Time		Q_b	*P*	*k*	*d*	*p*	95% CI
Language	Post-interven.	English	1.21	0.272	11	0.04	0.536	[-0.09, 0.17]
		German			5	0.17	0.071	[-0.01, 0.34]
Study goals	Post-interven.	*Health is…*						
		…only goal	1.20	0.274	11	0.15	0.003	[0.05, 0.24]
		…one of several goals or secondary goal			5	0.02	0.892	[-0.20, 0.23]
Voluntariness of participation	Post-interven.	Likely voluntary	1.82	0.177	12	0.04	0.583	[-0.10, 0.17]
		Involuntary			4	0.18	0.023	[0.03, 0.34]
Sample with health impairments	Post-interven.	Yes	0.44	0.506	5	0.14	0.133	[-0.04, 0.33]
		No			11	0.06	0.351	[-0.07, 0.20]
Randomi-zation	Post-interven.	Yes	1.78	0.183	6	0.01	0.953	[-0.17, 0.18]
		No			10	0.16	0.024	[0.02, 0.29]
Treatment control group	Post-interven.	No alternative treatment	0.65	0.419	10	0.05	0.594	[-0.14, 0.23]
		Alt. treatment or standard training for unemp. ppl.			6	0.14	0.010	[0.03, 0.24]

Note. k = number of effect sizes; *d* = average meta-analytic effect size; CI= 95% confidence interval for *d*; Q_b = heterogeneity between groups; *p* = significance level for *d* or Q_b.

SUPPLEMENTARY ONLINE MATERIAL - C

List of primary studies used in meta-analysis

Caplan , R. D., Vinokur, A. D., Price, R. H., & Van Ryn, M. (1989). Job seeking, reemployment, and mental health: A randomized field experiment in coping with job loss. *Journal of Applied Psychology, 74*(5), 759-769. https://doi.org/10.1037/0021-9010.74.5.759

Carlier, B. E., Schuring, M., & Burdorf, A. (2018). Influence of an interdisciplinary re-employment programme among unemployed persons with mental health problems on health, social participation and paid employment. *Journal of Occupational Rehabilitation, 28*(1), 147-157. https://doi.org/10.1007/s10926-017-9704-3

Creed, P. A., Bloxsome, T. D., & Johnston, K. (2001). Self-esteem and self-efficacy outcomes for unemployed individuals attending occupational skills training programs. *Community, Work & Family, 4*(3), 285-303. https://doi.org/10.1080/01405110120089350

Creed, P. A., Hicks, R. E., & Machin, M. A. (1998). Behavioural plasticity and mental health outcomes for long-term unemployed attending occupational training programmes. *Journal of Occupational and Organizational Psychology, 71*(2), 171-191. https://doi.org/10.1111/j.2044-8325.1998.tb00671.x

Creed, P. A., Machin, M. A., & Hicks, R. E. (1999). Improving mental health status and coping abilities for long-term unemployed youth using cognitive-behaviour therapy based training interventions. *Journal of Organizational Behaviour*, 20, 963-978. https://doi.org/10.1002/(SICI)1099-1379(199911)20:6<963::AID-JOB916>3.0.CO;2-D

Dambrun, M., & Dubuy, A.-L. (2014). A positive psychology intervention among long-term unemployed people and its effects on psychological distress and well-being. *Journal of Employment Counseling, 51*, 75-88. https://doi.org/10.1002/j.2161-1920.2014.00043.x

De Jong, A., Hommes, M., Brouwers, A. & Tomic, W. (2013). Effects of mindfulness-based stress reduction course on stress, mindfulness, job self-efficacy and motivation among unemployed people. *Australian Journal of Career Development, 22*(2), 51-62. https://doi.org/10.1177/1038416213486095

Gabrys, L., Michallik, L., Thiel, C., Vogt, L., & Banzer, W. (2013). Effects of a structured physical-activity counseling and referral scheme in long-term unemployed individuals: a pilot accelerometer study. *Behavioral Medicine, 39*(2), 44-50. https://doi.org/10.1080/08964289.2013.782852

Hanewinkel, R., Wewel, M., Stephan, C., Isensee, B., & Wiborg, G. (2006). Motivational interviewing of unemployed – acceptance and effects of counselling to improve health-related behavior. *Gesundheitswesen, 68*(4), 240-248. https://doi.org/10.1055/s-2006-926693

Harris, E., Lum, J., Rose, V., Morrow, M., Comino, E., & Harris, M. (2002). Are CBT interventions effective with disadvantaged job-seekers who are long-term unemployed? *Psychology, Health & Medicine, 7*(4), 401-410. https://doi.org/10.1080/13548500210000015221

Harry, J. M., & Tiggemann, M. (Mai 1992). The psychological impact of work re-entry training for female unemployed sole parents. *Australian Journal of Social Issues, 27*(2), 75-91. https://doi.org/10.1002/j.1839-4655.1992.tb01279.x

Herbig, B., Glaser, J., & Angerer, P. (2012). Old, sick, unemployed, without a chance? Results of a randomized controlled trial of the effects of a combined health and employment

promotion program for the older long-term unemployed. *Bundesgesundheitsblatt - Gesundheitsforschung - Gesundheitsschutz, 55*(8), 970-979. https://doi.org/10.1007/s00103-012-1514-3

Himle, J. A., Bybee, D., Steinberger, E., Laviolette, W. T., Weaver, A., Vlnka, S., . . . O'Donnell, L. A. (2014). Work-related CBT versus vocational services as usual for unemployed persons with social anxiety disorder: A randomized controlled pilot trial. *Behaviour Research and Therapy*, 169-176. https://doi.org/10.1016/j.brat.2014.10.005

Hodzic, S., Ripoll, P., Bernal, C., & Zenasni, F. (2015). The effects of emotional competences training among unemployed adults: A longitudinal study. *Applied Psychology: Health and Well-Being, 7*(3), 275-292. https://doi.org/10.1111/aphw.12048

Hong, P. Y., Choi, S., & Hong, R. (2020). A randomized controlled trial study of transforming impossible into possible (TIP) policy experiment in South Korea. *Research on Social Work Practice, 30*(6), 587-596. https://doi.org/10.1177/1049731519894647

Horns, K., Seeger, K., Heinmüller, M., Limm, H., Waldhoff, H.-P., Salman, R., Gündel, H., & Angerer, P. (2012). Health promotion for long-term unemployed. Effects on motivation for a healthy lifestyle. *Bundesgesundheitsblatt, 55,* 728–738. https://doi.org/10.1007/s00103-012-1477-4

Hulshof, I. L., Demerouti, E., & Le Blanc, P. M. (2020). A job search demands-resources intervention among the unemployed: Effects on well-being, job search behaviour and reemployment chances. *Journal of Occupational Health Psychology, 25*(1), 17-31. https://doi.org/10.1037/ocp0000167

Joseph, L. M., & Greenberg, M. A. (2001). The effects of a career transition program on reemployment success in laid-off professionals. *Consulting Psychology Journal: Practice and Research, 53*(3), S. 169-181. https://doi.org/10.1037/1061-4087.53.3.169

Kellner, A. (2021). Arbeitsförderung mit gesundheitsbezogener Ausrichtung – AmigA seit 2008 im Jobcenter MAIA des Landkreises Potsdam-Mittelmark. In: Hollederer, A. (Hrsg.), *Gesundheitsförderung bei Arbeitslosen* (pp. 318-333). Frankfurt am Main: Fachhochschulverlag. https://doi.org/

Limm, H., Heinmüller, M., Gündel, H., Liel, K., Seeger, K., Salman, R., & Angerer, P. (2015). Effects of a heatlh promotion program based on a train-the-trainer approach on quality of life and mental health of long-term unemployed persons. *BioMed Research International, Volume 2015*, pp. 1-10. https://doi.org/10.1155/2015/719327

Möllmann, C. (2009). *Evaluation einer gesundheitsbezogenen Maßnahme - Arbeitsförderung mit gesundheitsbezogener Ausrichtung*. Abschlussbericht zum Projekt AmigA+. Berlin.

Machin, M. A., & Creed, P. A. (2003). Understanding the differential benefits of training for the unemployed. *Australian Journal of Psychology, 55,* 104-113. https://doi.org/10.1080/00049530412331312964

Maguire, N., Hughes, V. C., Bell, L., Bogosian, A., & Hepworth, C. (2014). An evaluation of the choices for well-being project. *Psychology, Health & Medicine, 19*(3), 303-315. https://doi.org/10.1080/13548506.2013.806813

Maysent, A. M. (1995). *Coping with job loss and career stress: Effectiveness of stress management training with outplaced employees.* In Murphy, L. R., Hurrell, J. J., Sauter, Jr., S. L., & Keita, G. P. (Hrsg.), *Job stress interventions* (pp. 159–170). American Psychological Association. https://doi.org/10.1037/10183-011

Merakou, K., Tsoukas, K., Stavrinos, G., Amanaki, E., Daleziou, A., Kourmousi, N., . . . Barbouni, A. (2019). The Effect of progressive muscle relaxation on emotional competence: Depression–anxiety–stress, sense of coherence, health-related quality of

life, and well-being of unemployed people in Greece: An intervention study. *Explore, 15*(1), 38-46. https://doi.org/10.1016/j.explore.2018.08.001

Muller, J. (1992). The effects of personal training on the psychological state of long-term unemployed women. *Australian Psychologist, 27*(3), 176-180. https://doi.org/10.1080/00050069208257607

Paver, R., De Witte, H., Rothmann, S., Van den Broeck, A., & Blonk, R. W. B. (2020). The implementation and evaluation of the South African adaptation of the JOBS program. *Frontiers Psychology, 11,* 1-13. https://doi.org/10.3389/fpsyg.2020.01418

Proudfoot, J., Guest, D., Carson, J., Dunn, G., & Gray, J. (1997). Effect of cognitive-behavioural training on job-finding among long-term unemployed people. *Lancet, 350*(9071), 96-100. https://doi.org/10.1016/S0140-6736(96)09097-6

Reynolds, C., Barry, M. M., & Gabhainn, S. N. (2010). Evaluating the impact of the winning new jobs programme on the re-employment and mental health of a mixed profile of unemployed people. *International Journal of Mental Health Promotion, 12*(2), 32-41. https://doi.org/10.1080/14623730.2010.9721812

Roemer, A., Sutton, A., Grimm, C., & Medvedev, O. N. (2021). Effectiveness of a low-dose mindfulness-based intervention for alleviating distress in young unemployed adults. *Stress & Health, 37*(2), 320-328. https://doi.org/10.1002/smi.2997

Rothländer, K., Mühlpfordt, S., & Richter, P. (2012). Evaluation of the health promotion program 'Active coping with unemployment (AktivA)'. *Zeitschrift für Gesundheitspsychologie, 20*(3), 115-127. https://doi.org/10.1026/0943-8149/a000070

Rüsch, N., Staiger, T., Waldmann, T., Dekoj, M. C., Brosch, T., Gabriel, L., Bahemann, A., Oexle, N., Klein, T., Nehf, L., & Becker, T. (2019). Efficacy of peer-led group program for unemployed people with mental health problems: Pilot randomized controlled trial. *International Journal of Social Psychiatry, 65*(4), 333-337. https://doi.org/o0i.o.1rg1/1707.1/10707/00207067464001984466171171

Saam, R. H., Wodtke, K. H. & Hains, A. A. (1995). A cognitive stress reduction program for recently unemployed managers. *The career development quarterly, 44*(1), 43-51. https://doi.org/10.1002/j.2161-0045.1995.tb00527.x

Schuring, M., Burdorf, A., Voorham, A. J., der Weduwe, K., & Mackenbach, J. P. (2009). Effectiveness of a health promotion programme for long-term unemployed subjects with health problems: A randomised controlled trial. *Journal of Epidemiology and Community Health, 63*(11), 893-899. https://doi.org/10.1136/jech.2008.080432

Vinokur, A. D., Price, R. H., & Schul, Y. (1995). Impact of the JOBS intervention on unemployed workers varying in risk for depression. *American Journal of Community Psychology, 23*(1), 39-74. https://doi.org/10.1007/BF02506922

Vinokur, A.D., van Ryan, M., Gramlich, E. M., & Price, R. H. (1991). Long-term follow-up and benefit-cost analysis of the JOBS program: A preventive intervention for the unemployed. *Journal of Applied Psychology, 76*(2), 213-219. https://doi.org/10.1037/0021-9010.76.2.213

Vuori, J., & Silvonen , J. (2005). The benefits of a preventive job search program on re-employment and mental health at 2-year follow up. *Journal of Occupational and Organizational Psychology, 78*(1), 43-52. https://doi.org/10.1348/096317904X23790

SUPPLEMENTARY ONLINE MATERIAL - D: Forest plots depicting each primary study's effect size and confidence interval

Forest Plot - Mental Health - Post-Intervention

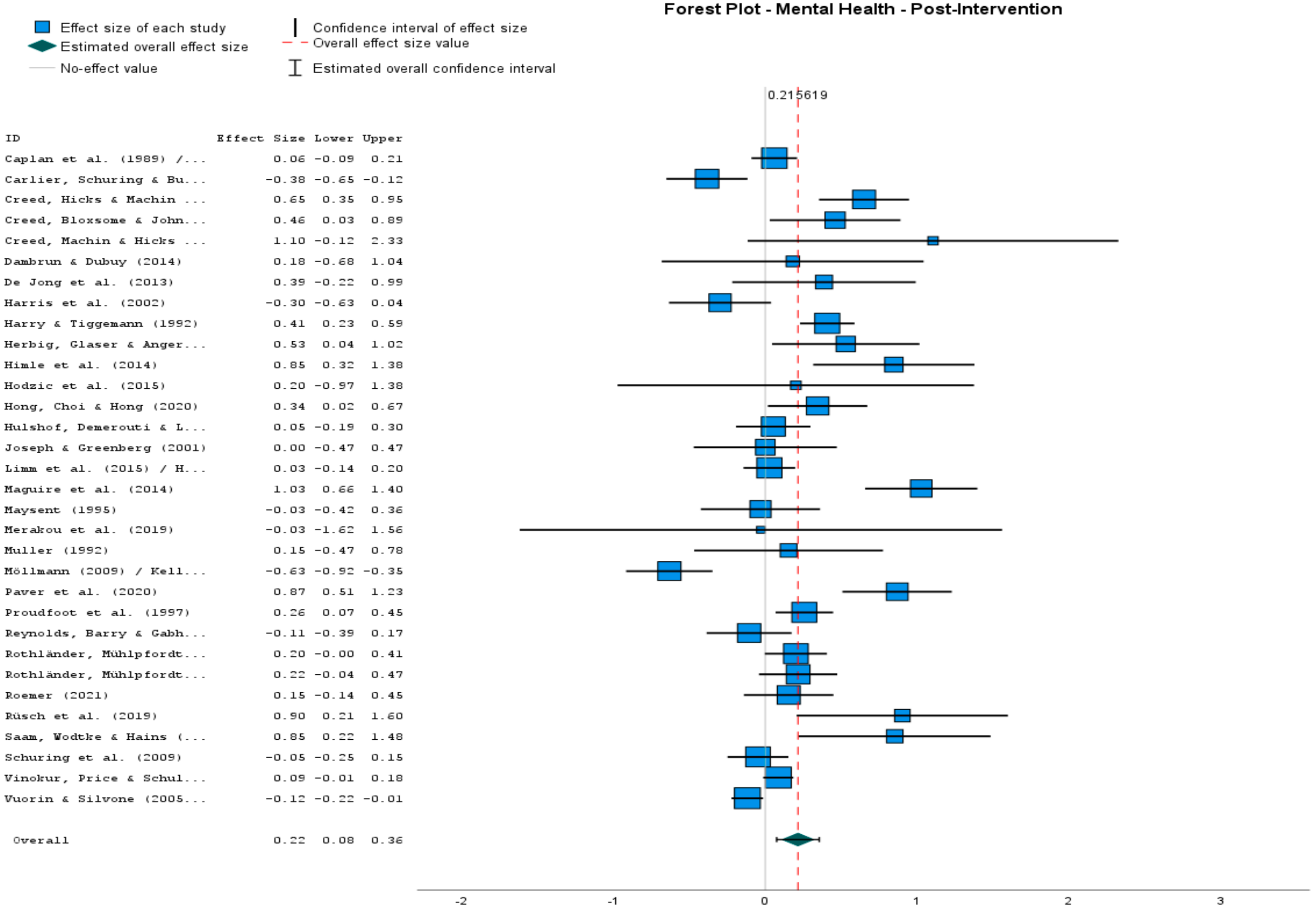

ID	Effect Size	Lower	Upper
Caplan et al. (1989) /...	0.06	-0.09	0.21
Carlier, Schuring & Bu...	-0.38	-0.65	-0.12
Creed, Hicks & Machin ...	0.65	0.35	0.95
Creed, Bloxsome & John...	0.46	0.03	0.89
Creed, Machin & Hicks ...	1.10	-0.12	2.33
Dambrun & Dubuy (2014)	0.18	-0.68	1.04
De Jong et al. (2013)	0.39	-0.22	0.99
Harris et al. (2002)	-0.30	-0.63	0.04
Harry & Tiggemann (1992)	0.41	0.23	0.59
Herbig, Glaser & Anger...	0.53	0.04	1.02
Himle et al. (2014)	0.85	0.32	1.38
Hodzic et al. (2015)	0.20	-0.97	1.38
Hong, Choi & Hong (2020)	0.34	0.02	0.67
Hulshof, Demerouti & L...	0.05	-0.19	0.30
Joseph & Greenberg (2001)	0.00	-0.47	0.47
Limm et al. (2015) / H...	0.03	-0.14	0.20
Maguire et al. (2014)	1.03	0.66	1.40
Maysent (1995)	-0.03	-0.42	0.36
Merakou et al. (2019)	-0.03	-1.62	1.56
Muller (1992)	0.15	-0.47	0.78
Möllmann (2009) / Kell...	-0.63	-0.92	-0.35
Paver et al. (2020)	0.87	0.51	1.23
Proudfoot et al. (1997)	0.26	0.07	0.45
Reynolds, Barry & Gabh...	-0.11	-0.39	0.17
Rothländer, Mühlpfordt...	0.20	-0.00	0.41
Rothländer, Mühlpfordt...	0.22	-0.04	0.47
Roemer (2021)	0.15	-0.14	0.45
Rüsch et al. (2019)	0.90	0.21	1.60
Saam, Wodtke & Hains (...	0.85	0.22	1.48
Schuring et al. (2009)	-0.05	-0.25	0.15
Vinokur, Price & Schul...	0.09	-0.01	0.18
Vuorin & Silvone (2005...	-0.12	-0.22	-0.01
Overall	0.22	0.08	0.36

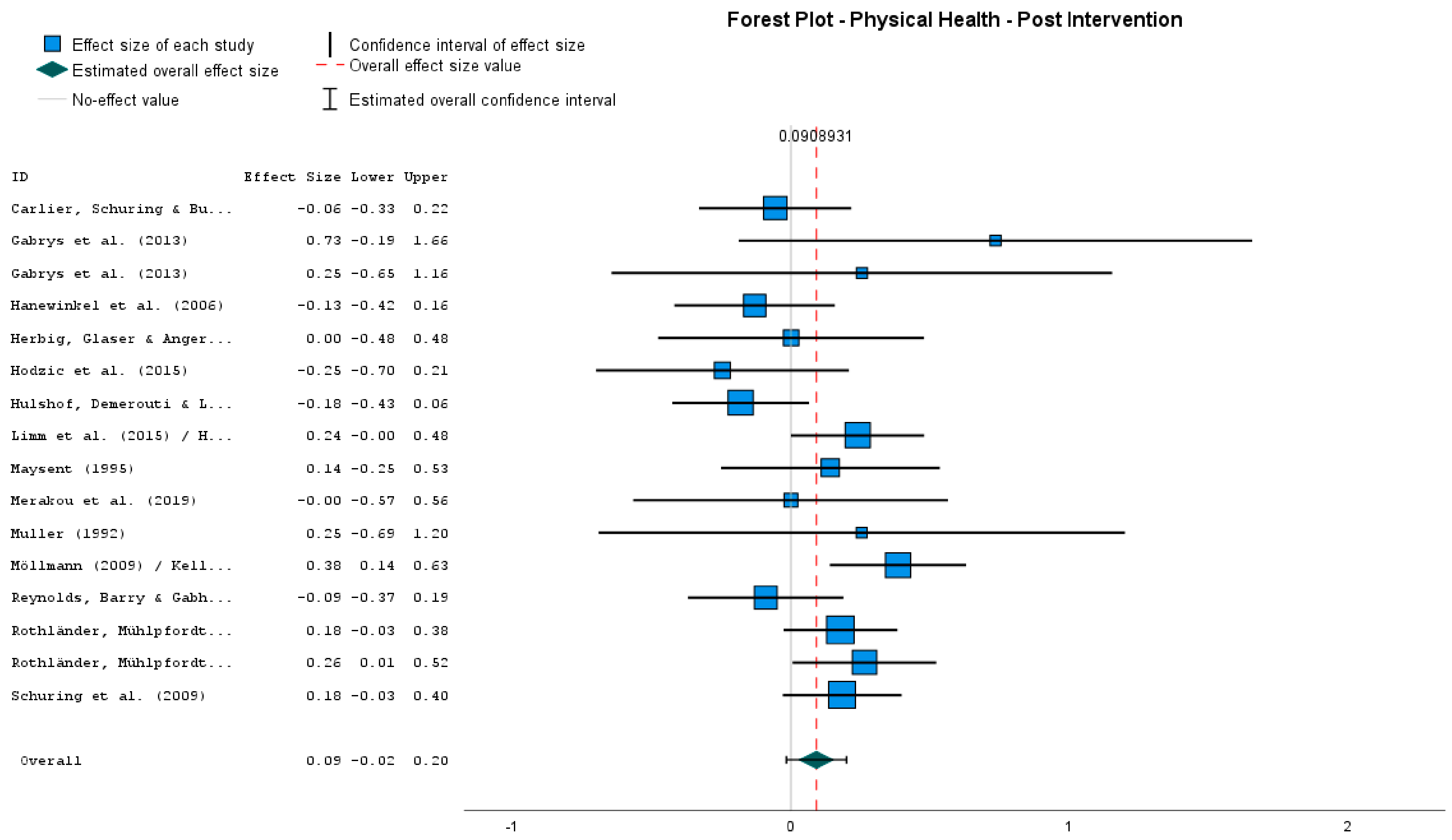
Forest Plot - Physical Health - Post Intervention
Effect size of each study
Estimated overall effect size
No-effect value
Confidence interval of effect size
Overall effect size value
Estimated overall confidence interval
0.0908931
ID Effect Size Lower Upper
Carlier, Schuring & Bu... -0.06 -0.33 0.22
Gabrys et al. (2013) 0.73 -0.19 1.66
Gabrys et al. (2013) 0.25 -0.65 1.16
Hanewinkel et al. (2006) -0.13 -0.42 0.16
Herbig, Glaser & Anger... 0.00 -0.48 0.48
Hodzic et al. (2015) -0.25 -0.70 0.21
Hulshof, Demerouti & L... -0.18 -0.43 0.06
Limm et al. (2015) / H... 0.24 -0.00 0.48
Maysent (1995) 0.14 -0.25 0.53
Merakou et al. (2019) -0.00 -0.57 0.56
Muller (1992) 0.25 -0.69 1.20
Möllmann (2009) / Kell... 0.38 0.14 0.63
Reynolds, Barry & Gabh... -0.09 -0.37 0.19
Rothländer, Mühlpfordt... 0.18 -0.03 0.38
Rothländer, Mühlpfordt... 0.26 0.01 0.52
Schuring et al. (2009) 0.18 -0.03 0.40
Overall 0.09 -0.02 0.20
-1
0
1
2

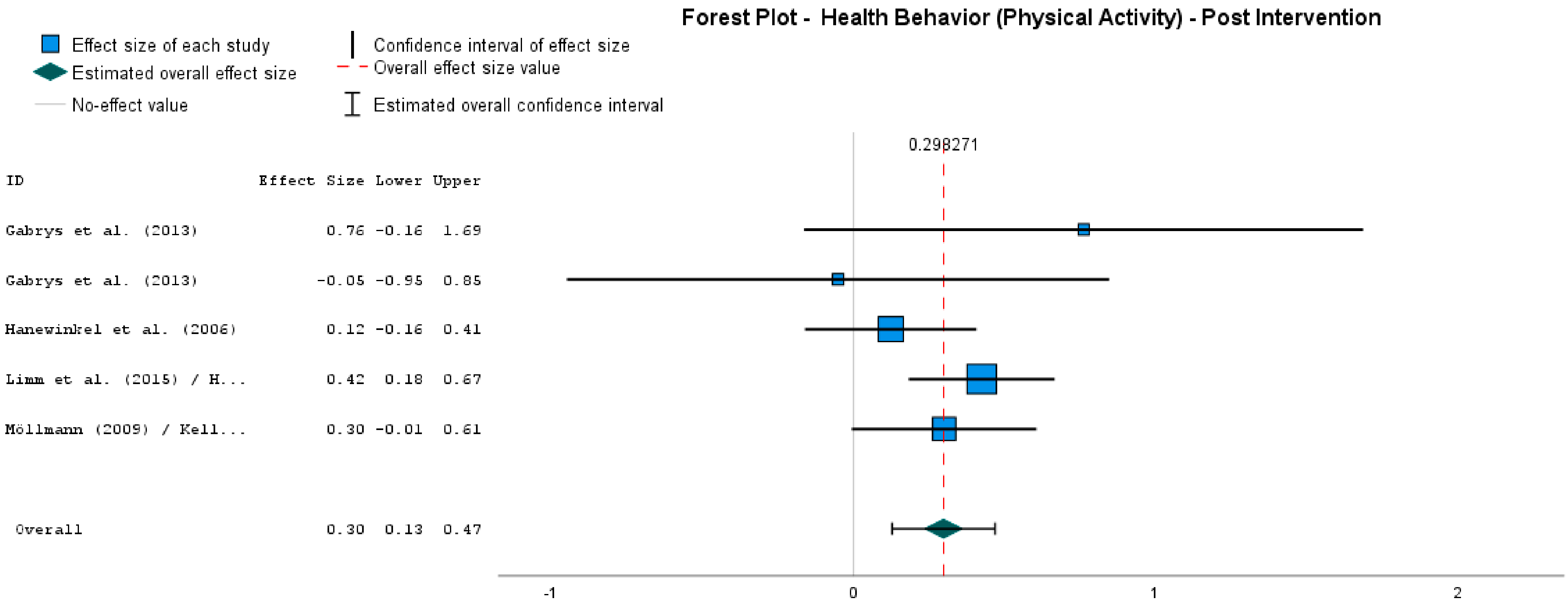

Forest Plot - Health Behavior (Physical Activity) - Post Intervention
Effect size of each study
Estimated overall effect size
No-effect value
Confidence interval of effect size
Overall effect size value
Estimated overall confidence interval
0.298271
ID
Effect Size Lower Upper
Gabrys et al. (2013) 0.76 -0.16 1.69
Gabrys et al. (2013) -0.05 -0.95 0.85
Hanewinkel et al. (2006) 0.12 -0.16 0.41
Limm et al. (2015) / H... 0.42 0.18 0.67
Möllmann (2009) / Kell... 0.30 -0.01 0.61
Overall 0.30 0.13 0.47
-1
0
1
2

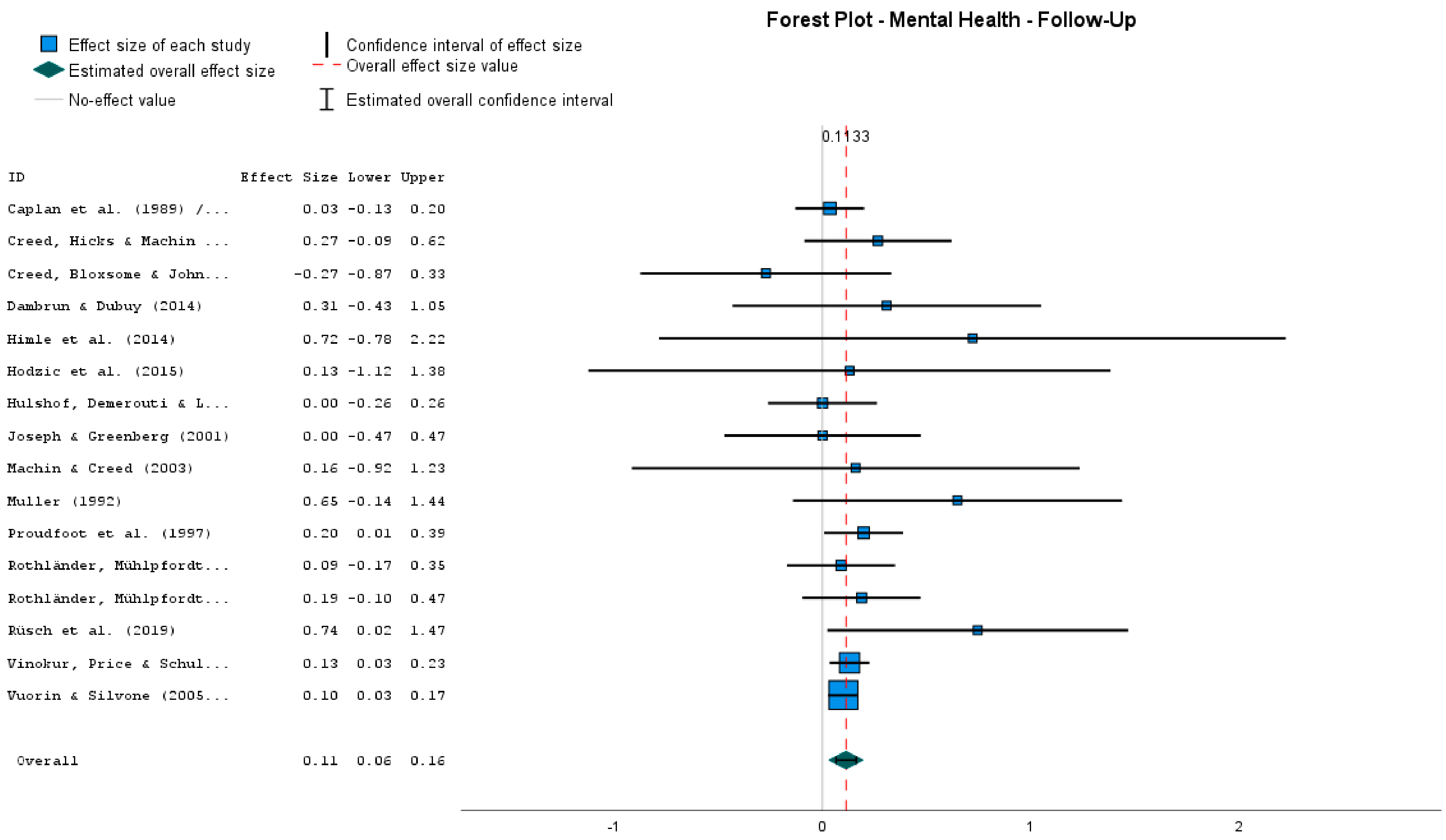
Forest Plot - Mental Health - Follow-Up
Effect size of each study
Estimated overall effect size
No-effect value
Confidence interval of effect size
Overall effect size value
Estimated overall confidence interval
0.1133
ID Effect Size Lower Upper
Caplan et al. (1989) /... 0.03 -0.13 0.20
Creed, Hicks & Machin ... 0.27 -0.09 0.62
Creed, Bloxsome & John... -0.27 -0.87 0.33
Dambrun & Dubuy (2014) 0.31 -0.43 1.05
Himle et al. (2014) 0.72 -0.78 2.22
Hodzic et al. (2015) 0.13 -1.12 1.38
Hulshof, Demerouti & L... 0.00 -0.26 0.26
Joseph & Greenberg (2001) 0.00 -0.47 0.47
Machin & Creed (2003) 0.16 -0.92 1.23
Muller (1992) 0.65 -0.14 1.44
Proudfoot et al. (1997) 0.20 0.01 0.39
Rothländer, Mühlpfordt... 0.09 -0.17 0.35
Rothländer, Mühlpfordt... 0.19 -0.10 0.47
Rüsch et al. (2019) 0.74 0.02 1.47
Vinokur, Price & Schul... 0.13 0.03 0.23
Vuorin & Silvone (2005... 0.10 0.03 0.17
Overall 0.11 0.06 0.16
-1
0
1
2

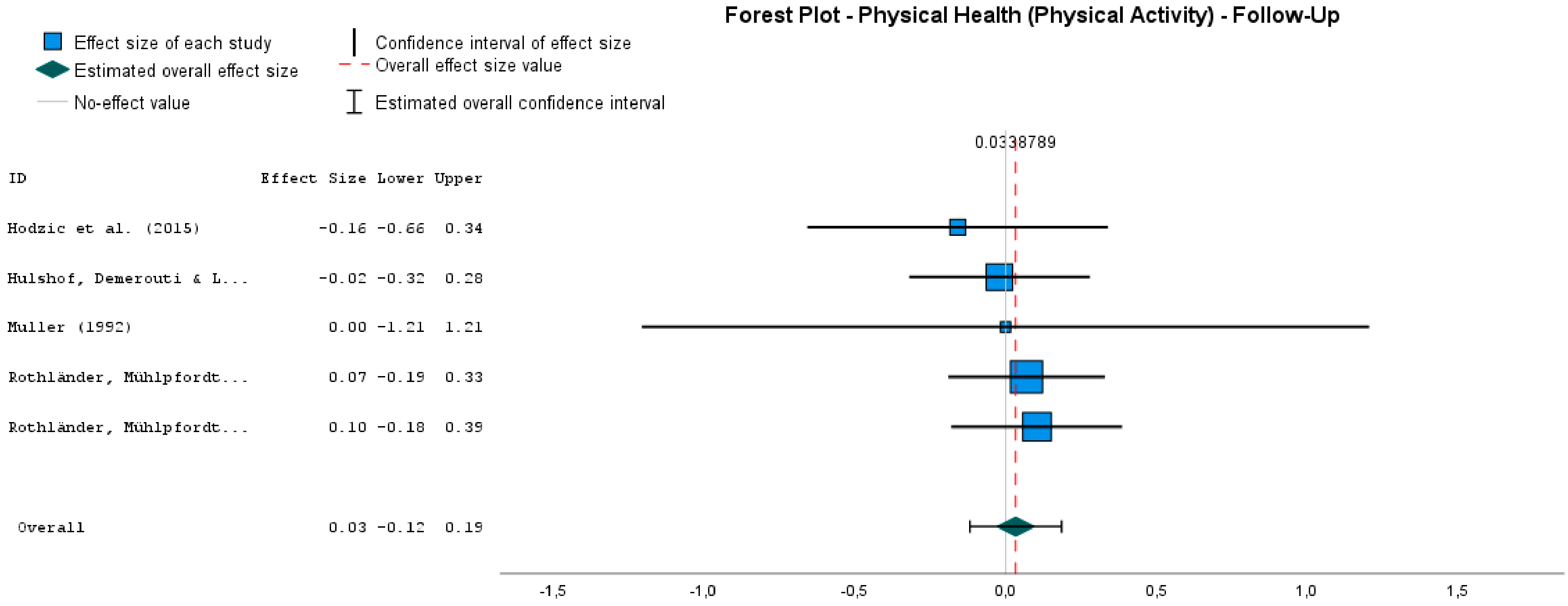

Forest Plot - Physical Health (Physical Activity) - Follow-Up
Effect size of each study
Estimated overall effect size
No-effect value
Confidence interval of effect size
Overall effect size value
Estimated overall confidence interval
0.0338789
ID
Effect Size Lower Upper
Hodzic et al. (2015) -0.16 -0.66 0.34
Hulshof, Demerouti & L... -0.02 -0.32 0.28
Muller (1992) 0.00 -1.21 1.21
Rothländer, Mühlpfordt... 0.07 -0.19 0.33
Rothländer, Mühlpfordt... 0.10 -0.18 0.39
Overall 0.03 -0.12 0.19
-1,5
-1,0
-0,5
0,0
0,5
1,0
1,5

Codings of formal characteristics of intervention & demographics

	Duration of intervention (weeks)	Intensity of intervention (number of hours average participant received treatment)	Number of participants per training group	Percentage of women in sample	Average age of the sample	Average years of formal education	mean duration of unemployment
Caplan et al. (1989)	2,00	24,00	18	54,00	35,90	12,90	3,00
Carlier et al. (2017)	104,29	-88,00	1	55,07	40,50	-88,00	53,63
Creed et al. (1998)	5,50	220,00	-88	45,86	33,00	-88,00	-88,00
Creed et al. (1999)	,43	15,00	-88	46,00	19,00	-88,00	-88,00
Creed et al. (2001)	5,00	200,00	-88	55,56	27,73	-88,00	10,87
Dambrun & Dubuy (2014)	2,00	1,00	1	52,38	41,00	-88,00	15,41
De Jong et al. (2013)	8,00	20,00	20	60,00	46,67	13,12	-88,00
Gabrys et al. (2013)-PAC	2,21	-88,00	1	-88,00	54,94	-88,00	64,96
Gabrys et al. (2013)-PAC + Exercise	14,21	-88,00	1	-88,00	54,73	-88,00	47,78
Hanewinkel et al. (2006)	,14	1,00	1	41,59	44,39	12,09	29,51
Harris et al. (2002)	,29	11,00	7	50,00	35,00	-88,00	39,00
Harry & Tiggemann (1992)	3,29	136,00	10	100,00	35,00	-88,00	-88,00
Herbig et al. (2012)	25,98	10,97	1	52,11	53,34	9,85	46,65
Himle et al. (2014)	4,00	16,00	4	32,80	43,59	11,71	-88,00
Hodzic et al. (2015)	2,42	15,00	10	86,60	34,37	-88,00	16,89
Hong et al. (2016)	1,20	20,00	8	53,25	49,85	-88,00	-88,00
Hulshof et al. (2020)	6,29	14,00	12	56,51	50,77	-88,00	15,92
Joseph & Greenberg (2001)	2,00	3,00	-88	40,00	46,80	-88,00	2,30
Limm et al. (2015)/Horns et al. (2012)	12,99	37,50	-88	56,10	44,10	10,50	68,63

Machin & Creed (2003)	,29	16,00	8	81,32	33,99	11,08	42,17
Maguire et al. (2014)	5,00	80,00	9	59,70	-88,00	-88,00	28,24
Maysent (1995)	1,00	6,00	-88	8,85	46,67	-88,00	-88,00
Merakou et al. (2018)	8,60	27,40	7	54,00	33,52	12,07	36,00
Möllmann (2009)/Kellner (2021)	-88,00	-88,00	-88	45,98	41,54	9,69	54,68
Muller (1992)	1,00	40,00	11	100,00	38,00	-88,00	132,00
Paver et al. (2020)	1,00	20,00	18	60,62	30,76	10,70	36,40
Proudfoot et al. (1997)	7,00	21,00	13	17,00	43,00	-88,00	24,58
Reynolds et al. (2010)	1,50	20,00	17	58,80	33,83	11,99	47,96
Roemer et al. (2017)	4,00	4,00	-88	35,60	20,03	-88,00	-88,00
Rothländer et al. (2012) - freely accessible	3,00	24,00	-88	62,26	49,56	13,49	20,39
Rothländer et al. (2012) - integrated	3,00	24,00	-88	60,13	45,23	12,88	22,97
Rüsch et al. (2019)	6,00	8,00	-88	52,00	46,10	13,50	46,30
Saam et al. (1995)	4,00	5,50	1	19,05	46,00	-88,00	-88,00
Schuring et al. (2009)	12,00	108,00	17	49,16	41,22	11,08	48,54
Vinokur et al. (1995)	1,00	20,00	16	55,00	36,20	13,95	,95
Vuori & Silvonen (2005)	1,00	20,00	10	77,80	37,00	-88,00	10,70

SUPPLEMENTARY ONLINE MATERIAL - E2: Tables with codings of study characteristics

Coding of study characteristics

	Language of study report	Importance of health as goal of intervention	Level of voluntariness of participation	Sample with health impairments	Randomization dichotomous	Treatment control group
Caplan et al. (1989)	English	Health one of several goals or secondary goal	high level of voluntarines	Sample has preexisting health impairments	randomization	no training/waiting list/booklet
Carlier et al. (2017)	English	Health one of several goals or secondary goal	low level of voluntariness	No health impairments	no randomization	course or training for unemployed or spec. designed alt. treatment
Creed et al. (1998)	English	Health one of several goals or secondary goal	high level of voluntarines	Sample has preexisting health impairments	no randomization	no training/waiting list/booklet
Creed et al. (1999)	English	Health only goal	high level of voluntarines	Sample has preexisting health impairments	no randomization	no training/waiting list/booklet
Creed et al. (2001)	English	Health one of several goals or secondary goal	high level of voluntarines	Sample has preexisting health impairments	no randomization	no training/waiting list/booklet
Dambrun & Dubuy (2014)	English	Health only goal	high level of voluntarines	Sample has preexisting health impairments	no randomization	course or training for unemployed or spec. designed alt. treatment
De Jong et al. (2013)	English	Health one of several goals or secondary goal	high level of voluntarines	Sample has preexisting health impairments	randomization	no training/waiting list/booklet
Gabrys et al. (2013)-PAC	English	Health only goal	high level of voluntarines	Sample has preexisting health impairments	no randomization	no training/waiting list/booklet
Gabrys et al. (2013)-PAC + Exercise	English	Health only goal	high level of voluntarines	Sample has preexisting health impairments	no randomization	no training/waiting list/booklet
Hanewinkel et al. (2006)	German	Health only goal	high level of voluntarines	Sample has preexisting health impairments	randomization	no training/waiting list/booklet

Harris et al. (2002)	English	Health one of several goals or secondary goal	high level of voluntarines	Sample has preexisting health impairments	randomization	course or training for unemployed or spec. designed alt. treatment
Harry & Tiggemann (1992)	English	Health one of several goals or secondary goal	high level of voluntarines	Sample has preexisting health impairments	randomization	no training/waiting list/booklet
Herbig et al. (2012)	German	Health one of several goals or secondary goal	high level of voluntarines	No health impairments	randomization	no training/waiting list/booklet
Himle et al. (2014)	English	Health one of several goals or secondary goal	high level of voluntarines	No health impairments	randomization	course or training for unemployed or spec. designed alt. treatment
Hodzic et al. (2015)	English	Health only goal	high level of voluntarines	Sample has preexisting health impairments	randomization	no training/waiting list/booklet
Hong et al. (2016)	English	Health one of several goals or secondary goal	high level of voluntarines	Sample has preexisting health impairments	randomization	no training/waiting list/booklet
Hulshof et al. (2020)	English	Health one of several goals or secondary goal	high level of voluntarines	Sample has preexisting health impairments	randomization	no training/waiting list/booklet
Joseph & Greenberg (2001)	English	Health one of several goals or secondary goal	high level of voluntarines	Sample has preexisting health impairments	randomization	course or training for unemployed or spec. designed alt. treatment
Limm et al. (2015)/Horns et al. (2012)	English	Health only goal	high level of voluntarines	Sample has preexisting health impairments	randomization	course or training for unemployed or spec. designed alt. treatment
Machin & Creed (2003)	English	Health only goal	high level of voluntarines	Sample has preexisting health impairments	randomization	course or training for unemployed or spec. designed alt. treatment
Maguire et al. (2014)	English	Health one of several goals or secondary goal	high level of voluntarines	No health impairments	randomization	no training/waiting list/booklet
Maysent (1995)	English	Health only goal	high level of voluntarines	Sample has preexisting health impairments	no randomization	no training/waiting list/booklet
Merakou et al. (2018)	English	Health only goal	high level of voluntarines	No health impairments	no randomization	no training/waiting list/booklet
Möllmann (2009)/Kellner (2021)	German	Health one of several goals or secondary goal	low level of voluntariness	No health impairments	no randomization	no training/waiting list/booklet

Muller (1992)	English	Health only goal	high level of voluntarines	Sample has preexisting health impairments	no randomization	no training/waiting list/booklet
Paver et al. (2020)	English	Health one of several goals or secondary goal	high level of voluntarines	Sample has preexisting health impairments	randomization	no training/waiting list/booklet
Proudfoot et al. (1997)	English	Health one of several goals or secondary goal	high level of voluntarines	Sample has preexisting health impairments	randomization	course or training for unemployed or spec. designed alt. treatment
Reynolds et al. (2010)	English	Health one of several goals or secondary goal	high level of voluntarines	Sample has preexisting health impairments	no randomization	course or training for unemployed or spec. designed alt. treatment
Roemer et al. (2017)	English	Health only goal	low level of voluntariness	Sample has preexisting health impairments	randomization	course or training for unemployed or spec. designed alt. treatment
Rothländer et al. (2012) - freely accessible	German	Health only goal	high level of voluntarines	Sample has preexisting health impairments	no randomization	course or training for unemployed or spec. designed alt. treatment
Rothländer et al. (2012) - integrated	German	Health only goal	low level of voluntariness	Sample has preexisting health impairments	no randomization	course or training for unemployed or spec. designed alt. treatment
Rüsch et al. (2019)	English	Health one of several goals or secondary goal	high level of voluntarines	No health impairments	randomization	no training/waiting list/booklet
Saam et al. (1995)	English	Health one of several goals or secondary goal	high level of voluntarines	Sample has preexisting health impairments	randomization	course or training for unemployed or spec. designed alt. treatment
Schuring et al. (2009)	English	Health only goal	low level of voluntariness	No health impairments	randomization	course or training for unemployed or spec. designed alt. treatment
Vinokur et al. (1995)	English	Health one of several goals or secondary goal	high level of voluntarines	Sample has preexisting health impairments	randomization	no training/waiting list/booklet
Vuori & Silvonen (2005)	English	Health one of several goals or secondary goal	high level of voluntarines	Sample has preexisting health impairments	randomization	no training/waiting list/booklet

Coding of type of intervention

	Intervention includes cognitive Methods	Intervention includes non-cognitive stress management	Intervention includes relaxation techniques	Intervention includes methods to increase social support	Intervention includes teaching of job search methods	Intervention includes direct physical exercise	Intervention includes health counselling
Caplan et al. (1989)	No	yes	no	yes	yes	no	no
Carlier et al. (2017)	yes	no	no	no	yes	no	no
Creed et al. (1998)	no	no	no	no	yes	no	no
Creed et al. (1999)	yes	yes	no	no	no	no	no
Creed et al. (2001)	no	no	no	no	yes	no	no
Dambrun & Dubuy (2014)	no	yes	no	no	no	no	no
De Jong et al. (2013)	no	no	yes	no	no	yes	no
Gabrys et al. (2013)-PAC	no	no	no	no	no	no	yes
Gabrys et al. (2013)-PAC + Exercise	no	no	no	no	no	yes	yes
Hanewinkel et al. (2006)	no	no	no	no	no	no	yes
Harris et al. (2002)	yes	no	yes	no	no	no	no
Harry & Tiggemann (1992)	yes	no	no	no	yes	no	no
Herbig et al. (2012)	no	no	no	no	no	yes	yes
Himle et al. (2014)	yes	no	no	no	no	no	no
Hodzic et al. (2015)	no	yes	no	no	no	no	no
Hong et al. (2016)	no	yes	yes	yes	yes	no	no
Hulshof et al. (2020)	no	no	no	no	yes	no	no
Joseph & Greenberg (2001)	yes	no	yes	no	yes	no	no
Limm et al. (2015)/Horns et al. (2012)	no	no	no	no	no	yes	yes

Machin & Creed (2003)	yes	no	no	no	no	no	no
Maguire et al. (2014)	yes	no	no	no	yes	no	no
Maysent (1995)	yes	yes	yes	no	no	no	no
Merakou et al. (2018)	no	no	yes	no	no	no	no
Möllmann (2009)/Kellner (2021)	no	no	no	no	no	no	yes
Muller (1992)	no	yes	no	no	yes	no	no
Paver et al. (2020)	no	yes	no	yes	yes	no	no
Proudfoot et al. (1997)	yes	no	no	no	yes	no	no
Reynolds et al. (2010)	no	yes	no	yes	yes	no	no
Roemer et al. (2017)	no	no	yes	no	no	no	no
Rothländer et al. (2012) - freely accessible	yes	no	yes	yes	no	no	no
Rothländer et al. (2012) - integrated	yes	no	yes	yes	no	no	no
Rüsch et al. (2019)	yes	no	yes	yes	no	no	no
Saam et al. (1995)	yes	yes	no	no	no	no	no
Schuring et al. (2009)	yes	no	no	no	no	yes	yes
Vinokur et al. (1995)	no	yes	no	yes	yes	no	no
Vuori & Silvonen (2005)	no	yes	no	yes	yes	no	no
